# Effects of Electroacupuncture for Opioid-Induced Constipation in Patients With Cancer in China: A Randomized Clinical Trial

**DOI:** 10.1001/jamanetworkopen.2023.0310

**Published:** 2023-02-22

**Authors:** Weiming Wang, Yan Liu, Xiaofang Yang, Jianhua Sun, Zenghui Yue, Dianrong Lu, Kehua Zhou, Yuanjie Sun, Aihua Hou, Zhiwei Zang, Xiaoqing Jin, Chao Liu, Yuhang Wang, Jinna Yu, Lili Zhu, Zhishun Liu

**Affiliations:** 1 Department of Acupuncture and Moxibustion, Guang’anmen Hospital, China Academy of Chinese Medical Sciences, Beijing, China; 2 Key Laboratory of Chinese Internal Medicine of the Ministry of Education, Dongzhimen Hospital, Beijing University of Chinese Medicine, Beijing, China; 3 College of Acumox and Tuina, Guizhou University of Traditional Chinese Medicine, Guiyang, China; 4 Department of Acupuncture Rehabilitation, the Affiliated Hospital of Nanjing University of Chinese Medicine, Nanjing, China; 5 College of Acupuncture and Tuina, Hunan University of Chinese Medicine, Changsha, Hunan, China; 6 Department of Oncology, Wang Jing Hospital, China Academy of Chinese Medical Sciences, Beijing, China; 7 Department of Hospital Medicine, ThedaCare Regional Medical Center–Appleton, Appleton, Wisconsin; 8 Department of Oncology, Yantai Hospital of Traditional Chinese Medicine, Yantai, China; 9 Department of Acupuncture, Yantai Hospital of Traditional Chinese Medicine, Yantai, China; 10 Department of Acupuncture, Zhejiang Hospital, Hangzhou, China

## Abstract

**Importance:**

Opioid-induced constipation (OIC) is prevalent among patients treated with opioids for cancer pain. Safe and effective therapies for OIC in patients with cancer remain an unmet need.

**Objective:**

To determine the efficacy of electroacupuncture (EA) for OIC in patients with cancer.

**Design, Setting, and Participants:**

This randomized clinical trial was conducted at 6 tertiary hospitals in China among 100 adult patients with cancer who were screened for OIC and enrolled between May 1, 2019, and December 11, 2021.

**Interventions:**

Patients were randomized to receive 24 sessions of EA or sham electroacupuncture (SA) over 8 weeks and then were followed up for 8 weeks after treatment.

**Main Outcomes and Measures:**

The primary outcome was the proportion of overall responders, defined as patients who had at least 3 spontaneous bowel movements (SBMs) per week and an increase of at least 1 SBM from baseline in the same week for at least 6 of the 8 weeks of the treatment period. All statistical analyses were based on the intention-to-treat principle.

**Results:**

A total of 100 patients (mean [SD] age, 64.4 [10.5] years; 56 men [56.0%]) underwent randomization; 50 were randomly assigned to each group. Among them, 44 of 50 patients (88.0%) in the EA group and 42 of 50 patients (84.0%) in the SA group received at least 20 (≥83.3%) sessions of treatment. The proportion of overall responders at week 8 was 40.1% (95% CI, 26.1%-54.1%) in the EA group and 9.0% (95% CI, 0.5%-17.4%) in the SA group (difference between groups, 31.1 percentage points [95% CI, 14.8-47.6 percentage points]; *P* < .001). Compared with SA, EA provided greater relief for most OIC symptoms and improved quality of life among patients with OIC. Electroacupuncture had no effects on cancer pain and its opioid treatment dosage. Electroacupuncture-related adverse events were rare, and, if any, all were mild and transient.

**Conclusions and Relevance:**

This randomized clinical trial found that 8-week EA treatment could increase weekly SBMs with a good safety profile and improve quality of life for the treatment of OIC. Electroacupuncture thus provided an alternative option for OIC in adult patients with cancer.

**Trial Registration:**

ClinicalTrials.gov Identifier: NCT03797586

## Key Points

**Question:**

What is the efficacy and safety of electroacupuncture for opioid-induced constipation in adult patients with cancer pain?

**Findings:**

This randomized clinical trial that included 100 patients with cancer and opioid-induced constipation found that the proportion of overall responders was greater with electroacupuncture (40.1%) than with sham electroacupuncture (9.0%) at week 8, a significant difference.

**Meaning:**

Electroacupuncture treatment could increase weekly spontaneous bowel movements with a good safety profile.

## Teaser

This randomized clinical trial assesses the effect of electroacupuncture for opioid-induced constipation in patients with cancer in China.

## Introduction

Opioids are the cornerstone of cancer pain management,^1^ and they are used for pain relief at all stages of cancer treatments. Approximately 28% of all patients with cancer^2^ and 33% to 40% of cancer survivors have chronic pain,^3^ and most patients with chronic cancer pain are receiving long-term opioid treatment. Opioid-induced constipation (OIC) affects 60% to 90%^4^ of patients with cancer-related opioid use. Opioid-induced constipation can have a profound negative effect on the activities of daily living and overall quality of life^5^ of patients with moderate to severe cancer pain.^6^ It may result in opioid discontinuation or voluntary opioid dose reduction, leading to inadequate cancer pain control.^7^ Unlike many other opioid-related adverse events (AEs), OIC is often unavoidable, is rarely tolerated,^7^ and does not improve with adjustment of the opioid regimen.^8^

Treatments for OIC often include laxatives^9^ and stool softeners coupled with increased dietary fiber, fluid intake, and exercise. However, these interventions do not target the underlying pathophysiology of OIC^10^ and are associated with limited efficacy or may themselves cause AEs.^7,11,12,13^ Peripherally acting μ-opioid receptor antagonists, such as methylnaltrexone and naldemedine, are a novel class of drugs that block the peripheral gastrointestinal opioid receptors responsible for OIC without compromising opioid analgesic effects.^14^ Although these medications have an efficacy rate of 50% or more,^15,16,17^ their use remains limited in clinical practice due to their cost, adverse effects, and availability in local pharmacies.

Two randomized clinical trials have indicated that electroacupuncture (EA) was superior to sham electroacupuncture (SA) and noninferior to prucalopride in treating chronic severe functional constipation, with a good safety profile; the effects of EA were maintained even 24 weeks after treatment.^18,19^ A recent meta-analysis suggested that acupuncture might relieve constipation and improve quality of life for patients with OIC^20^; however, the evidence was very low quality because the included studies had inherent flaws. Hence, we performed this multicenter, randomized clinical trial to evaluate the efficacy and safety of EA for OIC in adult patients with cancer pain.

## Methods

### Study Design and Participants

This was a multicenter, sham-controlled, randomized clinical trial performed between May 1, 2019, and December 11, 2021, at 6 hospitals across China. The trial protocol (Supplement 1) was approved by the ethics committee of all participating sites (Guang’anmen Hospital affiliated with China Academy of Chinese Medical Sciences, Guizhou University of Traditional Chinese Medicine, The Affiliated Hospital of Nanjing University of Chinese Medicine, Hunan University of Chinese Medicine, Wangjing Hospital affiliated with China Academy of Chinese Medical Sciences, Yantai Hospital of Traditional Chinese Medicine, and Zhejiang Hospital). The trial was conducted according to the Declaration of Helsinki^21^ and the Good Clinical Practice guidance and followed the Consolidated Standards of Reporting Trials (CONSORT) reporting guideline. Written informed consent was obtained from each patient before participation.

Patients 18 to 85 years of age were screened for the following (must have all for inclusion): diagnosis of histologically confirmed malignant neoplasm, receiving stable cancer treatment(s), life expectancy of 6 months or longer, and receiving an opioid regimen with a total daily dose of 30 to 1000 mg of oral morphine equivalent for at least 2 weeks. Patients were included in the trial if they had received a diagnosis of OIC according to the Rome IV criteria,^22^ had fewer than 3 spontaneous bowel movements (SBMs) per week, and had an Eastern Cooperative Oncology Group performance status of 0 to 3 at baseline. Key exclusion criteria included constipation due to reasons other than opioids (eg, irritable bowel syndrome) (eTable 1 in Supplement 2). After a 1-week run-in period without the use of laxatives or stool softeners, patients were randomly assigned to receive either EA or SA through a centralized web-based randomization system at a ratio of 1:1 using permuted block randomization. Patients, outcome assessors, data managers, and the statistician (Y.L.) were blinded to group allocation, while acupuncturists were not.

### Intervention

The EA treatment and SA control protocols were based on previous clinical trials^18,19^ and expert consensus. All acupuncturists were licensed and had a minimum of 2 years’ clinical experience and received standardized training prior to trial initiation.

Patients in both groups received 24 sessions of treatment, 30 minutes each session, over an 8-week period (3 sessions each week, ideally every other day), and were followed up for 8 weeks thereafter. For the EA group, bilateral acupoints Tianshu (ST25), Fujie (SP14), and Shangjuxu (ST37) (eFigure in Supplement 2) were used. With patients in the supine position and after skin disinfection, acupuncturists inserted 0.30 × 50-mm or 0.30 × 75-mm disposable needles vertically into acupoints ST25 and SP14 for approximately 30 to 70 mm until reaching the muscle layer of the abdominal wall (acupuncturists feel the resistance from the needle tip) and inserted 0.30 × 40-mm needles vertically at bilateral acupoint ST37 for approximately 15 mm. Then, acupuncturists performed gentle manipulations (small equal lifting and twisting) at the needle handles 3 times for all acupoints to reach *de qi* (a sensation described as soreness, heaviness, swelling, or numbness).^23^ Paired alligator clips of the EA apparatus (SDZ-V; Suzhou Medical Appliance Factory) were attached transversely to the needle handles at bilateral acupoints ST25, SP14, and ST37, with a continuous wave of 10 Hz and a current intensity of 0.5 to 4 mA, depending on the patient’s comfort level. For the SA group, needles were inserted vertically 2 to 3 mm at nonacupoints (eFigure in Supplement 2) without needle manipulation; electrodes were then attached with a current intensity of 0.1 to 0.2 mA for 30 seconds. Treatment protocols were otherwise the same for the 2 groups.

During the trial, we discouraged patients from receiving other interventions for OIC. However, the use of bisacodyl suppository (5-10 mg; maximum of 20 mg/d) and/or a 110-mL glycerol enema as rescue medication was permitted if the patient had not had a bowel movement for 72 hours or more during the trial. Details of rescue medicine and other interventions if used were recorded in the stool diary.

Before treatment, we told patients that they had a 50% chance of receiving conventional EA vs minimal EA (SA) with possible similar efficacy, and they may or may not feel the stimulation during treatment because of the relatively low stimulation intensity. Patients were treated separately to avoid communication. To assess the success of blinding, within 5 minutes after treatment at week 8, patients were asked to guess whether they had received conventional EA.

### Outcomes

The primary outcome was the proportion of overall responders, defined as those having at least 3 SBMs per week and an increase from baseline of at least 1 SBM per week for at least 6 of the 8 weeks of treatment. An SBM was defined as a bowel movement occurring without the use of any rescue medicine or other interventions in the previous 24 hours.^24^ We also analyzed the proportions of sustained responders, defined as patients who fulfilled these criteria for at least 3 of the last 4 weeks of treatment (post hoc).^25^

Secondary outcomes included change from baseline in the mean weekly SBMs and complete SBMs (CSBMs; defined as SBMs with the feeling of complete evacuation) during weeks 1 to 8 and weeks 13 to 16; the proportion of patients with 3 or more mean weekly SBMs and CSBMs, and those with an increase of 1 or more mean weekly SBM and CSBMs from baseline during weeks 1 to 8 and weeks 13 to 16; change from baseline in the mean Bristol Stool Form Scale score (range, 1-7 indicating different stool consistency, where 1 indicates separate hard lumps and 7 indicates entirely liquid)^26^ and in the mean score for straining (range, 0-4, where 0 indicates not difficult and 4 indicates extremely difficult) during weeks 1 to 8 and weeks 13 to 16; change from baseline in the total and subscale scores of the Patient Assessment of Constipation–Symptoms (PAC-SYM) questionnaire (range, 0-4, where 0 indicates best outcomes and 4 indicates worst outcomes, and 1.0 is considered the minimal clinically important difference)^27^ and the Chinese-version Patient Assessment of Constipation Quality of Life (PAC-QOL) questionnaire (range, 0-4, where 0 indicates best outcomes and 4 indicates worst outcomes, and 1.0 is considered the minimal clinically important difference)^27^ at weeks 8 and 16; patients’ global assessment of therapeutic effects (range, 1-7, where 1 indicates markedly worse and 7 indicates markedly improved) at weeks 8 and 16; and the proportion and mean frequency of patients using rescue medicine and other interventions during the trial period.

Patients’ expectations of acupuncture for general illness and OIC were assessed at baseline. Safety assessments included AEs, changes in opioid dosage, and cancer pain score. All AEs were classified by acupuncturists and oncologists within 24 hours of their occurrence as treatment related or nontreatment related. Changes in opioid dosage were monitored and captured as the number of patients with a 30% or greater weekly mean increase or decrease in opioid dosage from baseline, and cancer pain was rated using the Numerical Rating Scale (range, 0-10, where 0 indicates no pain and 10 indicates the worst imaginable pain) in the preceding week.

### Statistical Analysis

Based on the results of our pilot study (unpublished), a 14% responder rate in the SA group was assumed in this trial. With a dropout rate of approximately 15%, a sample size of 100 patients was estimated to provide 90% power to detect a between-group difference of 31.4 percentage points at a 2-sided *P* < .05 significance level.

The primary outcome was assessed by fitting a generalized linear model with a binomial distribution and identity link. The same approach was used for other categorical variables, such as the proportion of patients with 3 or more mean weekly SBMs and CSBMs and those with an increase of 1 or more mean weekly SBMs and CSBMs from baseline, proportions of patients using rescue medicine, and other interventions. Similar models were used for subgroup analyses based on baseline daily opioid dose (preplanned: 30-100 mg vs >100 mg oral morphine equivalent) and type of primary cancer (post hoc: lung cancer vs nonlung cancer).

Changes from baseline in mean SBMs and CSBMs during weeks 1 to 8 and weeks 13 to 16 were analyzed by fitting linear mixed-effects models using treatment, visit, and treatment-by-visit interaction as fixed effects. The same approach was used for other continuous variables, such as the PAC-SYM score and the PAC-QOL score. Patients’ global response assessment, blinding, adherence, and AE data were provided for descriptive purposes only.

The intention-to-treat population included all participants who were randomized according to randomized treatment assignment. Missing data on the primary outcome were imputed using the multiple imputation method under the missing at random assumption.^28^ To assess the robustness of the primary analyses, we conducted 4 sensitivity analyses (eAppendix in Supplement 2).

All statistical analyses were performed according to the intention-to-treat principle using SAS, version 9.4 (SAS Institute Inc), with a 2-sided *P* < .05 considered statistically significant. No adjustment was made for multiple comparisons; therefore, secondary outcomes should be interpreted as exploratory.

## Results

**Figure 1.**
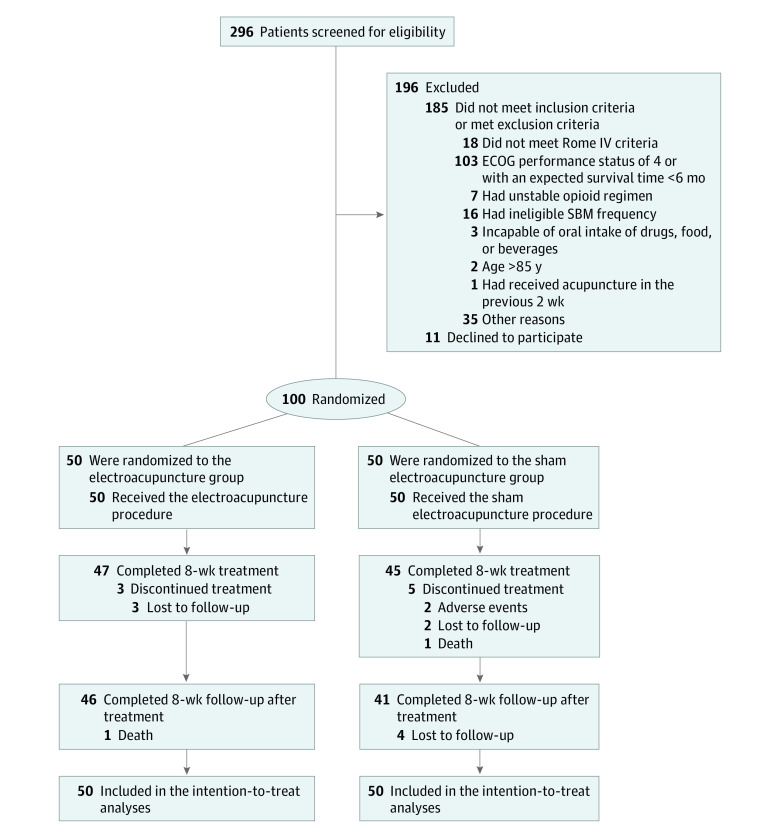
Study Flow Diagram ECOG indicates Eastern Cooperative Oncology Group; and SBM, spontaneous bowel movement.

Between May 1, 2019, and December 11, 2021, 100 patients (mean [SD] age, 64.4 [10.5] years; 56 men [56.0%]) were randomly assigned to either the EA group (n = 50) or the SA group (n = 50) (Figure 1). During the 8-week treatment, stool diary data were partially missing for 3 patients (6.0%) in the EA group and 5 patients (10.0%) in the SA group. Baseline characteristics, including expectations of acupuncture, were similar between groups (Table 1; eTable 2 in Supplement 2).

**Table 1.** Baseline Characteristics

Characteristic	Patients, No. (%)	
	Electroacupuncture (n = 50)	Sham electroacupuncture (n = 50)
Age, mean (SD), y	63.6 (10.4)	65.1 (10.6)
Sex		
Male	29 (58.0)	27 (54.0)
Female	21 (42.0)	23 (46.0)
Educational level		
High school diploma or less	41 (82.0)	40 (80.0)
Associate degree	6 (12.0)	8 (16.0)
Bachelor degree	3 (6.0)	2 (4.0)
BMI, mean (SD)	21.9 (2.7)	21.9 (3.2)
Occupational status		
Employed	4 (8.0)	4 (8.0)
Retired	22 (44.0)	20 (40.0)
Other	24 (48.0)	26 (52.0)
Marital status		
Married	48 (96.0)	46 (92.0)
Unmarried	2 (4.0)	0
Widowed	0	4 (8.0)
ECOG performance status^a^		
0	0	1 (2.0)
1	3 (6.0)	7 (14.0)
2	36 (72.0)	22 (44.0)
3	11 (22.0)	20 (40.0)
Type of primary cancer		
Lung	17 (34.0)	21 (42.0)
Liver	6 (12.0)	4 (8.0)
Breast	5 (10.0)	3 (6.0)
Pancreas	4 (8.0)	2 (4.0)
Uterine cervix	2 (4.0)	3 (6.0)
Nasopharynx	3 (6.0)	1 (2.0)
Colorectal	2 (4.0)	1 (2.0)
Stomach	2 (4.0)	1 (2.0)
Other^b^	9 (18.0)	14 (28.0)
Time since the primary cancer diagnosis, mean (SD), mo	22.4 (23.6)	23.0 (22.0)
Patients with cancer metastasis on enrollment	21 (42.0)	27 (54.0)
Type of treatment received at baseline		
Radiotherapy	3 (6.0)	8 (16.0)
Chemotherapy	33 (66.0)	19 (38.0)
Chemotherapy plus radiotherapy	5 (10.0)	8 (16.0)
Targeted therapy	7 (14.0)	11 (22.0)
Other	2 (4.0)	4 (8.0)
Type of opioid administered		
Oxycodone	25 (50.0)	39 (78.0)
Morphine	10 (20.0)	6 (12.0)
Other^c^	15 (30.0)	5 (10.0)
Opioid dose		
Mean duration of current opioid use (SD), wk	14.0 (10.7)	20.8 (26.2)
Mean total daily dose of opioid at baseline (SD), mg^d^	84.2 (83.8)	73.5 (50.9)
No. of patients stratified by opioid dose		
30-100 mg	37 (74.0)	38 (76.0)
>100 mg	13 (26.0)	12 (24.0)
Time since OIC diagnosis, mean (SD), mo	2.9 (2.3)	4.3 (5.3)
History of chronic functional constipation	0	4 (8.0)
Comorbidities		
Hypertension	14 (28.0)	15 (30.0)
Diabetes	7 (14.0)	5 (10.0)
Other	12 (24.0)	11 (22.0)
No. of SBMs/wk, mean (SD)	1.6 (0.5)	1.5 (0.5)
No. of CSBMs/wk, mean (SD)	0.4 (0.5)	0.3 (0.6)
Stool consistency score by Bristol stool scale, mean (SD)^e^	2.2 (0.6)	2.2 (0.8)
Defecation straining score, mean (SD)^f^	2.9 (0.5)	2.8 (0.7)
PAC-SYM, mean (SD)		
Total score^g^	2.3 (0.8)	2.3 (0.7)
Stool symptoms score	2.9 (0.7)	2.9 (0.7)
Rectal symptoms score	2.4 (0.9)	2.4 (0.8)
Abdominal symptoms score	1.7 (0.9)	1.6 (0.9)
PAC-QOL total score, mean (SD)^h^	2.2 (0.8)	2.3 (0.8)
Dissatisfied with previous treatment, No./total No. (%)^i^	23/24 (95.8)	21/25 (84.0)
Patients who used rescue medicine at baseline		
Rescue medicine	24 (48.0)	26 (52.0)
Other rescue treatment^j^	3 (6.0)	4 (8.0)
Frequency of rescue medicine used per week at baseline, mean (SD)		
Rescue medicine	2.0 (1.6)	2.9 (3.8)
Other rescue treatment^j^	4.7 (4.7)	5.0 (3.5)
NRS score for cancer-related pain intensity at baseline, mean (SD)^k^		
Mean cancer intensity	3.1 (1.5)	3.4 (1.6)
Worst cancer intensity	4.7 (1.5)	4.9 (1.5)

Abbreviations: BMI, body mass index (calculated as weight in kilograms divided by height in meters squared); CSBM, complete spontaneous bowel movement; ECOG, Eastern Cooperative Oncology Group; NRS, Numerical Rating Scale; OIC, opioid-induced constipation; PAC-QOL, Patient Assessment of Constipation Quality of Life; PAC-SYM, Patient Assessment of Constipation–Symptoms; SBM, spontaneous bowel movement.

^a^ Values for ECOG performance status range from 0 to 5; higher scores indicate greater disability.

^b^ Other types of cancer in the electroacupuncture group include kidney cancer (2 cases) and bile duct cancer, testis cancer, bone cancer, lymphoid cancer, ovarian cancer, tongue cancer, and esophageal cancer (1 case of each type). Other types of cancer in the sham electroacupuncture group include prostate cancer (3 cases); lymphoid cancer (2 cases); and gallbladder cancer, bile duct cancer, soft-tissue sarcoma cancer, osteosarcoma cancer, ovarian cancer, esophageal cancer, penis cancer, bladder cancer, and ureter cancer (1 case of each type).

^c^ Other opioids administered include fentanyl (3 in the electroacupuncture group and 2 in the sham group), hydrocodone (1 in each treatment group), tramadol (11 in the electroacupuncture group and 1 in the sham group), and codeine (1 in the sham group).

^d^ Oral morphine equivalent.

^e^ Scores range from 1 to 7: 1 = separate, hard lumps, like nuts (hard to pass); 2 = sausage-shaped but lumpy; 3 = like a sausage or snake but with cracks on its surface; 4 = like a sausage or snake, smooth and soft; 5 = soft blobs with clear-cut edges (passed easily); 6 = fluffy pieces with ragged edges or a mushy stool; 7 = watery, no solid pieces (entirely liquid).

^f^ Scores range from 0 to 4; higher scores indicate more severe straining during defecation.

^g^ PAC-SYM scores are based on a 5-point Likert scale from 0 to 4; lower scores indicate better quality of life.

^h^ PAC-QOL scores are based on a 5-point Likert scale from 0 to 4; lower scores indicate better quality of life.

^i^ Data were based on patients with treatments for OIC in the previous week.

^j^ Manual maneuvers to facilitate defecations (1 in the sham electroacupuncture group) and Chinese herbs (3 in each group).

^k^ The NRS is an 11-point scale from 0 to 10; 0 indicates no pain and 10 indicates the maximum pain imaginable.

The mean (SD) number of treatment sessions was 21.7 (4.1) in the EA group and 21.0 (6.2) in the SA group; 44 of 50 patients (88.0%) in the EA group and 52 of 50 (84.0%) in the SA group received at least 20 (≥83.3%) sessions of treatment (eTable 3 in Supplement 2). The proportion of overall responders during treatment was significantly higher with EA than SA (40.1% [95% CI, 26.1%-54.1%] vs 9.0% [95% CI, 0.5%-17.4%]; between-group difference, 31.1 percentage points [95% CI, 14.8-47.6 percentage points]; *P* < .001) (Table 2).^27^ The 4 sensitivity analyses (eTable 4 in Supplement 2) and 2 subgroup analyses (eTable 5 in Supplement 2) yielded similar outcomes. Coincidentally, the proportions of sustained responders were essentially the same for the 2 groups as the primary outcome (Table 2).^27^

**Table 2.** Primary and Secondary Outcomes

Variable	Electroacupuncture (n = 50)	Sham electroacupuncture (n = 50)	Difference, percentage points (95% CI)	*P* value
**Primary outcome**				
Overall responders, % (95% CI)^a^	40.1 (26.1 to 54.1)	9.0 (0.5 to 17.4)	31.1 (14.8 to 47.6)	<.001
**Secondary outcomes**				
Sustained responders, No./total No. (%)^b^	19/47 (40.4)	4/45 (8.9)	31.5 (15.2 to 47.9)	<.001
Changes in the mean weekly SBMs, mean (95% CI)				
Weeks 1-8	1.2 (1.0 to 1.3)	0.6 (0.4 to 0.8)	0.6 (0.3 to 0.8)	<.001
Weeks 13-16	0.6 (0.4 to 0.8)	0.2 (0.04 to 0.4)	0.4 (0.1 to 0.6)	.01
Patients with ≥3 mean weekly SBMs, No./total No. (%)				
Weeks 1-8	19/47 (40.4)	6/45 (13.3)	27.1 (9.9 to 44.3)	.002
Weeks 13-16	4/46 (8.7)	1/41 (2.4)	6.3 (−3.2 to 15.7)	.19
Patients with an increase of ≥1 mean weekly SBM, No./total No. (%)				
Weeks 1-8	30/47 (63.8)	14/45 (31.1)	32.7 (13.4 to 52.0)	<.001
Weeks 13-16	12/46 (26.1)	4/41 (9.8)	16.3 (0.7 to 31.9)	.04
Changes in mean weekly CSBMs, mean (95% CI), No.				
Weeks 1-8	0.8 (0.7 to 1.0)	0.4 (0.2 to 0.5)	0.2 (0.2 to 0.8)	<.001
Weeks 13-16	0.3 (0.1 to 0.5)	0.1 (−0.1 to 0.3)	0.2 (−0.1 to 0.5)	.17
Patients with ≥3 mean weekly CSBMs, No./total No. (%)				
Weeks 1-8	0	0	NA	NA
Weeks 13-16	1/46 (2.2)	0	NA	NA
Patients with an increase of ≥1 mean weekly CSBMs, No./total No. (%)				
Weeks 1-8	21/47 (44.7)	9/45 (20.0)	24.7 (6.3 to 43.1)	.009
Weeks 13-16	10/46 (21.7)	4/41 (9.8)	12.0 (−3.0 to 27.0)	.12
Change in stool consistency score by Bristol stool scale, mean (95% CI)^c^				
Weeks 1-8	0.5 (0.3 to 0.7)	0.3 (0.1 to 0.5)	0.2 (−0.1 to 0.6)	.14
Weeks 13-16	0.3 (0.01 to 0.5)	−0.1 (−0.3 to 0.2)	0.3 (−0.02 to 0.7)	.07
Change in score for defecation straining, mean (95% CI)^d^				
Weeks 1-8	−0.6 (−0.8 to −0.4)	−0.1 (−0.3 to 0.1)	−0.5 (−0.8 to −0.2)	.001
Weeks 13-16	−0.3 (−0.5 to −0.1)	0.04 (−0.2 to 0.3)	−0.3 (−0.7 to −0.02)	.04
Change in PAC-SYM total score, mean (95% CI)^e^				
Week 8	−0.9 (−1.0 to −0.7)	−0.5 (−0.7 to −0.4)	−0.4 (−0.6 to −0.2)	<.001
Week 16	−0.5 (−0.6 to −0.3)	−0.1 (−0.3 to 0.04)	−0.4 (−0.6 to −0.2)	.001
Change in PAC-SYM stool symptoms score, mean (95% CI)				
Week 8	−0.7 (−0.9 to −0.5)	−0.6 (−0.8 to −0.4)	−0.1 (−0.4 to 0.2)	.37
Week 16	−0.4 (−0.7 to −0.2)	−0.2 (−0.4 to 0.02)	−0.2 (−0.5 to 0.1)	.13
Change in PAC-SYM rectal symptoms score, mean (95% CI)				
Week 8	−1.0 (−1.2 to −0.8)	−0.6 (−0.7 to −0.4)	−0.4 (−0.7 to −0.3)	<.001
Week 16	−0.5 (−0.7 to −0.3)	−0.1 (−0.3 to 0.04)	−0.4 (−0.6 to −0.1)	.004
Change in PAC-SYM abdominal symptoms score, mean (95% CI)				
Week 8	−0.7 (−0.8 to −0.5)	−0.4 (−0.6 to −0.2)	−0.3 (−0.5 to 0.01)	.06
Week 16	−0.4 (−0.6 to −0.2)	−0.04 (−0.3 to 0.2)	−0.4 (−0.7 to −0.1)	.008
Change in PAC-QOL total score, mean (95% CI)^f^				
Week 8	−0.6 (−0.8 to −0.5)	−0.4 (−0.5 to −0.2)	−0.3 (−0.5 to −0.1)	.01
Week 16	−0.4 (−0.5 to −0.2)	−0.1 (−0.3 to 0.1)	−0.3 (−0.5 to −0.03)	.03
Patients with rescue medicine use, No./total No. (%)				
Weeks 1-8				
Rescue medicine	24/47 (51.1)	30/45 (66.7)	−15.6 (−35.5 to 4.2)	.12
Other rescue treatment^g^	5/47 (10.6)	4/45 (8.9)	1.8 (−10.4 to 13.9)	.77
Weeks 9-16				
Rescue medicine	28/46 (60.9)	29/41 (70.7)	−9.9 (−29.7 to 10.0)	.33
Other rescue treatment^h^	5/46 (10.9)	2/41 (4.9)	6.0 (−5.2 to 17.1)	.29
Frequency of rescue medicine use per week, mean (95% CI)				
Weeks 1-8				
Rescue medicine	0.4 (0.2 to 0.6)	0.7 (0.5 to 0.8)	−0.3 (−0.5 to 0.002)	.05
Other rescue treatment^g^	0.7 (0.2 to 1.3)	1.0 (0.3 to 1.5)	−0.2 (−1.1 to 0.6)	.50
Weeks 9-16				
Rescue medicine	0.6 (0.4 to 0.8)	0.9 (0.7 to 1.1)	−0.2 (−0.5 to 0.1)	.10
Other rescue treatment^h^	0.5 (−0.1 to 1.0)	0.47 (−0.4 to 1.3)	−0.01 (−1.0 to 1.0)	.99

Abbreviations: CSBM, complete spontaneous bowel movement; NA, not applicable; PAC-QOL, Patient Assessment of Constipation Quality of Life; PAC-SYM, Patient Assessment of Constipation–Symptoms; SBM, spontaneous bowel movement.

^a^ Overall responders were defined as patients who had at least 3 SBMs per week and an increase of at least 1 SBM from baseline in the same week for at least 6 of the 8 weeks of the treatment period.

^b^ Sustained responders were defined as patients who had at least 3 SBMs per week and an increase of at least 1 SBM from baseline in the same week for at least 6 of the 8 weeks of the treatment period and at least 3 of the final 4 treatment weeks (post hoc).

^c^ Scores range from 1 to 7: 1 = separate, hard lumps, like nuts (hard to pass); 2 = sausage-shaped but lumpy; 3 = like a sausage or snake but with cracks on its surface; 4 = like a sausage or snake, smooth and soft; 5 = soft blobs with clear-cut edges (passed easily); 6 = fluffy pieces with ragged edges or a mushy stool; 7 = watery, no solid pieces (entirely liquid).

^d^ Scores range from 0 to 4; higher scores indicate more severe straining during defecation.

^e^ PAC-SYM scores are based on a 5-point Likert scale from 0 to 4; lower scores indicate better quality of life. Minimally clinically important difference: 1.0.^27^

^f^ PAC-QOL scores are based on a 5-point Likert scale from 0 to 4; lower scores indicate better quality of life. Minimally clinically important difference: 1.0.^27^

^g^ Manual maneuvers to facilitate defecations (1 in the electroacupuncture group) and Chinese herbs (4 in each group).

^h^ Lactulose (2 in the electroacupuncture group), manual maneuvers to facilitate defecations (1 in the electroacupuncture group), and Chinese herbs (2 in each group).

**Figure 2.**
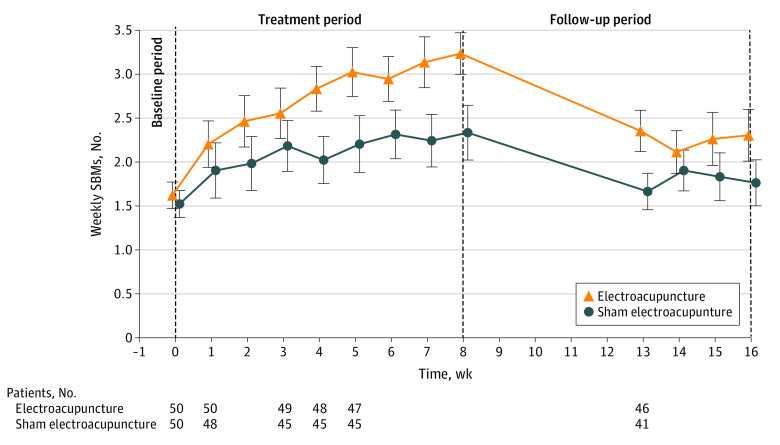
Weekly Spontaneous Bowel Movements (SBMs) During the Study Over Time, by Group The increase over baseline of weekly SBMs in the electroacupuncture group increased by 1 or more over baseline from week 4 and was maintained during weeks 4 to 8, whereas the increase over baseline of weekly SBMs in the sham electroacupuncture group was less than 1 throughout the study. Error bars indicate 95% CIs.

Patients in the EA group, as compared with the SA group, had a significant increase in the number of mean weekly SBMs from baseline over weeks 1 to 8 (between-group difference, 0.6 SBMs [95% CI, 0.3-0.8 SBMs]; *P* < .001) and weeks 13 to 16 (0.4 SBMs [95% CI, 0.1-0.6 SBMs]; *P* = .01) (Table 2).^27^ The increase in mean weekly SBMs over baseline was 1 or more in the EA group at week 4 and was maintained during weeks 4 to 8; however, it was less than 1 during weeks 13 to 16 in the EA group and for all assessments in the SA group (Figure 2; eTable 6 in Supplement 2). In addition, the EA group had better outcomes for many other secondary outcomes (Table 2^27^; eTable 7 and eTable 8 in Supplement 2). The between-group comparisons of weekly rescue medicine use favored the EA group for weeks 1 to 8 (between-group difference, –0.3 percentage points [95% CI, –0.5 to 0.002 percentage points]; *P* = .05) (Table 2),^27^ but not during weeks 9 to 16 (between-group difference, –0.2 percentage points [95% CI, –0.5 to 0.1 percentage points]; *P* = .10) (Table 2).^27^ For blinding assessment, 68 of 92 patients (73.9%) (46 in the EA group and 22 in the SA group) guessed that they received conventional EA. Nonetheless, in the SA group, the overall responder rates were similar between patients who guessed they received minimal EA (SA) and those who guessed they received conventional EA (eTable 9 in Supplement 2).

The mean and worst Numerical Rating Scale scores for cancer pain remained generally stable throughout the trial and had no meaningful between-group differences at weeks 8 and 16 (eTable 10 in Supplement 2). Similarly, the dosage of opioid use remained unchanged in both groups during assessments (eTable 11 in Supplement 2). Acupuncture-related AEs were transient and mild; serious AEs were rare and not related to EA (Table 3).

**Table 3.** Adverse Events

Adverse event	Patients, No. (%)	
	Electroacupuncture (n = 50)	Sham electroacupuncture (n = 50)
Any adverse event^a^	4 (8.0)	8 (16.0)
Serious adverse events^b^		
Death^c^	1 (2.0)	1 (2.0)
Cancer progression^d^	0	2 (4.0)
Acupuncture-related adverse events		
Local hematoma	2 (4.0)	3 (6.0)
Inconsequential bleeding	1 (2.0)	2 (4.0)

^a^ Adverse events were analyzed for all patients who received randomization. Types rather than frequencies of adverse events were counted for the same patient. Different types of adverse events occurring for a single patient were defined as independent adverse events. Adverse events occurring more than once for a single patient were counted as 1 adverse event.

^b^ A serious adverse event was defined as an adverse event that caused death, exacerbation of the preexisting condition, interruption of cancer treatment, prolongation of existing hospitalization, or permanent disability or damage, or required medical intervention to prevent 1 of the above outcomes.

^c^ Cancer progression (lung cancer in the electroacupuncture group and malignant lymphoma in the sham electroacupuncture group) rather than acupuncture was considered as the cause of death by the investigator.

^d^ Considered not related to acupuncture treatment by the investigator.

## Discussion

To our knowledge, this is the first multicenter randomized clinical trial to evaluate the efficacy of EA for OIC in patients with cancer. This trial demonstrated that 8-week EA, as compared with SA, is more likely to produce a significant and continuous increase in weekly SBMs. However, the between-group differences for mean weekly SBMs and CSBMs were less than 1, arguing against the clinical significance of EA over SA. Although EA had no effects on stool consistency, cancer pain severity, or opioid dosage, EA did decrease defecation straining and improve quality of life for patients with OIC.

The US Food and Drug Administration recommends overall responder analysis as the primary evaluation for gastrointestinal drug development.^29^ According to this recommendation and previous pharmaceutical trials for OIC medications,^30^ the responder analysis in this trial is a reasonable efficacy assessment for the effects of EA on OIC.^31^ The proportion of overall or sustained responders in the EA group was 40.1%, regardless of daily opioid dose. This finding was in line with a trial of naloxegol for OIC in patients with noncancer pain in which groups receiving 12.5 mg/d and 25 mg/d of naloxegol had sustained response rates of 40.8% and 44.4%, respectively, over a 12-week treatment period.^32^

The higher percentage of overall SBM responders in the EA group showed that EA was promising in normalizing bowel movements, which was also observed in a previous trial of EA for chronic functional constipation.^33^ This previous trial found that the percentage of overall CSBM responders with 8 weeks of EA was 26.6% after treatment and 39.0% during follow-up. However, in contrast to studies showing long-lasting effects for chronic functional constipation,^18,19,33^ the increase of mean weekly SBMs with EA decreased from 1 or more for weeks 4 to 8 to less than 1 for weeks 13 to 16. This discrepancy indicates that the efficacy of 8-week EA in the present trial is so small that it was not maintained after treatment, and continuous EA treatment might be necessary to maintain its benefits in normalizing bowel functions while patients are taking opioids. Another explanation could be that the pathophysiology of OIC is different from that of chronic functional constipation.

In the EA group, although the increase in weekly SBMs was 1 or more at week 4 and maintained through week 8, the increase in weekly CSBMs in the EA group was consistently less than 1 throughout the study. An increase of 1 or more CSBMs per week from baseline represents a clinically significant change,^34^ whereas the clinical effects of an increase of 1 or more weekly SBMs without a similar change in weekly CSBMs remain unclear. Complete SBM represents patients’ subjective feelings of complete evacuation. The placebo effect is an important component of EA efficacy^35^; however, this efficacy seemingly did not translate into patients’ subjective feelings of CSBM, especially given that the number of patients in the EA group who guessed they received EA was more than twice that of the SA group. In summary, in contrast to the previous trial that determined the benefit of EA in improving CSBM for chronic functional constipation,^33^ this trial did not indicate efficacy of EA in reducing an incomplete evacuation feeling for OIC in patients with cancer. The mechanisms underlying these differences warrant further exploration.

Relieving constipation symptoms and increasing constipation-associated well-being are the primary considerations when caring for individuals with an advanced illness such as cancer.^36,37^ This trial measured these considerations from a broader perspective using the PAC-SYM and the PAC-QOL. The results showed that EA was superior to SA for both assessments. In addition, a higher proportion of patients in the EA group than in the SA group reported moderate and marked improvements in the global assessment of therapeutic effects. The results of these assessments are in line with the primary outcome of this trial, favoring EA over SA.

Opioids cause constipation primarily through activation of the opioid receptors in the gastrointestinal tract,^38^ interfering with the normal tone and contractility of smooth muscles, delaying transit time of the fecal content.^39^ Wang et al^40^ found that EA was associated with increased gastric emptying and a shortened distal colon and whole-gut transit time via reversing sympathovagal imbalance in constipation caused by loperamide’s activation of opioid receptors in the myenteric plexus of the bowel. In addition, EA was also found to facilitate regeneration of the lost enteric neurons,^41^ and as 1 type of transcutaneous modulation, EA may modulate plasma levels of motilin, ghrelin, gastrin, and bile acid.^42^

### Limitations

This trial has some limitations. First, because of the wide heterogeneity of patients with cancer, the generalizability of this trial may be limited. Second, we measured the primary outcome using a self-recorded diary; within-individual variability might exist and introduce some bias. Third, acupuncturists could not be blinded, which may have resulted in inflated treatment outcomes. Fourth, the EA protocol remains to be optimized because approximately 60% of patients were nonresponders and the efficacy of EA decreased after 8 weeks of treatment. Fifth, the efficacy of EA is slow and may require coadministration of pharmaceuticals at the beginning of treatment.

## Conclusions

In this randomized clinical trial, an 8-week EA treatment exhibited a consistent and stable benefit with a good safety profile for OIC in adult patients with cancer. The effects of EA did not interfere with opioid analgesia. Electroacupuncture may be considered as an alternative for the management of OIC in patients with chronic cancer pain.

## Notes

Supplement 1.
Trial Protocol and Statistical Analysis Plan

Supplement 2. **eTable 1.** Methods: Trial Inclusion and Exclusion Criteria **eFigure.** Methods: Location of Acupoints for Both Study Arms **eAppendix.** Methods: Multiple Imputation and Sensitivity Analyses **eTable 2.** Results: Patients’ Expectations About the Effectiveness of Acupuncture at Baseline **eTable 3.** Results: Compliance Data at Week 8 **eTable 4.** Sensitivity Analyses for the Primary Outcome **eTable 5.** Results: Subgroup Analyses **eTable 6.** Results: Weekly SBMs **eTable 7.** Results: PAC-QOL Total and Subscale Scores **eTable 8.** Results: Patients’ Global Improvement Assessment, n (%) **eTable 9.** Results: Blinding Data and Their Association With the Primary Outcome by the Respective Group **eTable 10.** Results: Change From Baseline in NRS Score for Mean Cancer Pain Intensity and Worst Pain Intensity Within the Preceding Week **eTable 11.** Results: Proportion of Patients With Change From Baseline in the Dose of Opioid Consumption, n (%)
eReferences.

Supplement 3.
Data Sharing Statement
