# Supplementary material for: Effects of Electroacupuncture for Opioid-Induced Constipation in Patients With Cancer in China: A Randomized Clinical Trial
Source: JAMA Netw Open. 2023 Feb 22;6(2):e230310. doi: 10.1001/jamanetworkopen.2023.0310 (PMC9947731; doi:10.1001/jamanetworkopen.2023.0310)
Supplement: Supplement 1. — Trial Protocol and Statistical Analysis Plan [file jamanetwopen-e230310-s001.pdf]

This supplement contains the following items:

1. Final protocol, the summary of changes from the published original protocol

2. Final statistical analysis plan, the Summary of Changes of Final SAP.

Effects of electroacupuncture on opioid-induced constipation in patients with cancer: a multicenter randomized controlled trial

Clinical Sites:

1. Guang'an men Hospital Affiliated to China Academy of Chinese Medical Sciences
2. Guizhou University of Traditional Chinese Medicine
3. The Affiliated Hospital of Nanjing University of Chinese Medicine
4. Hunan University of Chinese Medicine
5. Wangjing Hospital Affiliated to China Academy of Chinese Medical Sciences
6. Yantai Hospital of Traditional Chinese Medicine
7. Zhejiang Hospital

Data Management and Statistical Centers:

Linkermed Pharm Technology Co. Ltd, Beijing, China

Data：

Original protocol date: September 30, 2018

Amendment date: November 29, 2019

Confidentiality Statement

This document is the intellectual property of the Investigators. The information provided in this document is strictly confidential and is available for review to the sponsor, investigators, potential investigators, appropriate Ethics Committees, Investigational Review Boards, and other government regulatory bodies. No disclosure should take place without written authorization from the protocol developing investigators, except to the extent necessary needed to obtain informed consent from potential subjects.

Table of Contents

1. Study Contact and Organization 2
   1.1 Study Contacts 2
   1.2 Recruiting Sites 3
   1.3 Collaborating Sites 5
2. Study Design 5
   2.1 Study Overview 5
   2.2 Background 5
   2.3 Study Hypothesis 7
   2.4 Methodology 7
   2.4.2 Subjects 8
   2.4.3 Trial flow chart 12
   2.4.4 Outcomes Measurement 15
3. Safety Assessment 18
4. Interventions 19
   4.1 EA 19
   4.2 SA 20
   4.3 Rescue medication 20
5. Informed consent 21
6. Quality Control 25
7. Data Management 26
   7.1 The Raw Data Management and Archiving 26
   7.2 Data Entry and Storage 26
   7.3 Data Verification and Problems Solving 27
   7.4 Medical Coding 27
   7.5 Data Report 27
   7.6 Data Auditing 28
   7.7 Database Locking 28
8. Statistical Consideration 28
   8.1 Statistical Analysis 29
   8.2 Statistical Analysis Plan (SAP) 29
9 Ethical principle 29
10 Funding 29
11 References 30
12 Major update of the published protocol 34

1 Study Contact and Organization

1.1 Study Contacts

Guang'anmen Hospital of China Academy of Chinese Medical Sciences:

Zhishun Liu, MD PhD (Principal Investigator for trial)
Director of Acupuncture and moxibustion Department
5 Beixian'ge Street, Xicheng District, Beijing, China, 100053
Phone: +86 10 88002331
Email: zhishunjournal@163.com

Weiming Wang, MD PhD
Acupuncture and moxibustion Department
5 Beixian'ge Street, Xicheng District, Beijing, China, 100053
Phone: +86 10 88002930
Email: wangweiming1a1@163.com

Yuanjie Sun, MD PhD
Acupuncture and moxibustion Department
5 Beixian'ge Street, Xicheng District, Beijing, China, 100053
Phone: +86 10 88001123
Email: puzhisun@163.com

Jinna Yu, MD PhD
Acupuncture and moxibustion Department
5 Beixian'ge Street, Xicheng District, Beijing, China, 100053
Phone: 13520566715
Email: 546872837@qq.com

Lili Zhu, MD
Acupuncture and moxibustion Department

5 Beixian'ge Street, Xicheng District, Beijing, China, 100053
Phone: 18851182160
Email: julius.zhu@foxmail.com

1.2 Recruiting Sites

Guizhou University of Traditional Chinese Medicine

Xiaofang Yang, MD PhD
Acumox and Tuina College
Dongqing South Road, Huaxi University Town, Guiyang, China, 550025
Phone: +86 13984096366
Email: yangxiaofang210@163.com

Chao Liu MD
Acumox and Tuina College
Dongqing South Road, Huaxi University Town, Guiyang, China, 550025
Phone: +86 18798089545
Email: 549889429@qq.com

The Affiliated Hospital of Nanjing University of Chinese Medicine

Jianhua Sun, MD PhD
Department of Acupuncture Rehabilitation
155 Hanzhong Road, Qinhuai District, Nanjing, Zhejiang, China, 210029
Phone: +86 13914722816
Email: 377201634@qq.com

Yuhang Wang, MD
Department of Acupuncture Rehabilitation
No.155, Hanzhong Road, Qinhuai District, Nanjing, Zhejiang, China, 210029
Phone: +86 18752018817
Email: wangyuhang@njucm.edu.cn

Hunan University of Chinese Medicine

Zenghui Yue, MD

College of Acupuncture and Tuina

300 Xueshi Road, Yuelu District, Changsha, Hunan, China, 410208

Phone: +86 13787293866

Email: 624755064@qq.com

Wangjing Hospital affiliated to China Academy of Chinese Medical Sciences

Dianrong Lu, MD PhD

Department of oncology

6 Wangjing Zhonghuan South street, Chaoyang District, Beijing, China, 100102

Phone: +86 13717998887

Email: ludianrong@aliyun.com

Yantai Hospital of Traditional Chinese Medicine

Aihua Hou, MD

Department of oncology

39 Xingfu Road, Zhifu District, Yantai, Shandong, China

Phone: +86 13001616877

E-mail: zlk2127926@163.com

Zhiwei Zang, MD

Department of Acupuncture and Massage

39 Xingfu Road, Zhifu District, Yantai, Shandong, China

Phone: +86 15553553797

E-mail: 860922902@qq.com

Zhejiang Hospital

Xiaoqing Jin, MD

Department of Acupuncture

No. 12 Lingyin Road, Hanzhou, Zhejiang, China, 310013

Phone: +86 13357102177
Email: zjyyjxq@163.com

1.3 Collaborating Sites

Dongzhimen Hospital, Beijing University of Chinese Medicine, Beijing, China
Yan Liu, MD
Key Laboratory of Chinese Internal Medicine of Ministry of Education
5 Haiyuncang Dongcheng, Beijing, China, 100700
Phone: +86 13811299493
Email: sasliu@yeah.net

Catholic Health System Internal Medicine Training Program, University at Buffalo, Buffalo, NY
Kehua zhou, MD, DPT
Phone: 716 986 4856
Email: kehuazho@buffalo.edu

2. Study Design

2.1 Study Overview

The objective of study is to assess the efficacy of electro-acupuncture (EA) for opioid-induced constipation(OIC) in adult patients with cancer pain.

2.2 Background

In advanced diseases, 70-80% of patients experience moderate to severe pain[1]. As the cornerstone of treatment for moderate to severe cancer pain, opiate analgesics, such as morphine and oxycodone, are recommended by WHO Cancer Pain Relief Guidelines [2-3]. The use of systemic opioids is recommended by some studies for cancer patients

experiencing moderate to severe pain, regardless of the underlying causes[4]. Opioids stimulate receptors both in the central nervous system (CNS) and the peripheral nervous system, reducing pain and improving quality of life for patients [5]. The drug can, however, be associated with serious adverse events (AEs) with a rate ranging from 1.8% to 13.6%[6-7], the most common of which is opioid-induced constipation (OIC). OIC represents a change in baseline bowel habits or defecation patterns that occurs following the administration or modification of opioid therapy [8-10]. Approximately 41% of non-cancer patients and 94% of cancer patients who use opioids for pain have this condition [11-12]. Symptoms of OIC are usually persistent and difficult to tolerate [9], which adversely affects patients' quality of life[8, 13-15] and results in reductions in dose or discontinuation of opioid analgesics [16]. OIC is the result of multiple factors contributing to it [17]: Opioids may activate μ-receptors throughout the gastrointestinal tract and cause changes to gut motility, decreases in gut secretion, and an increase in sphincter tone, which can lead to constipation [18]. Various pharmacological and nonpharmacological interventions are used to manage OIC, such as laxatives and increased fluid intake [8-9, 19]. However, these interventions are limited in effectiveness, and they do not address the pathophysiological mechanisms of OIC [8-9]. Several peripherally acting μ-opioid receptor antagonists (PAMORAs), such as naloxegol and methylnaltrexone, have recently been shown to be effective in treating OIC patients who do not respond to simple medications [20]. However, longer-term efficacy and safety of PAMORAs are unclear, and they haven't been approved in China yet. Clinical trials are still underway to test these drugs. Additionally, PAMORAs are often associated with AEs such as abdominal pain and flatulence[21]. As a result, it is still necessary to explore new treatment approaches for OIC.

In traditional Chinese medicine, acupuncture has been used to treat gastrointestinal disease, including constipation, for thousands of years. According to two systematic reviews, acupuncture can improve spontaneous bowel movements (SBMs) in functional constipation [22-23]. Additionally, the results of our study indicated that electroacupuncture (EA) could increase complete spontaneous bowel movements (CSBMs) and SBMs, with a long-term effect that continues for 24 weeks after treatment ceased among patients with chronic, severe functional constipation [24-25]. Through stimulation of the somatic and peripheral nervous systems, acupuncture can facilitate the gut motility and improve

gastrointestinal function [26]. The effectiveness of acupuncture for OIC is currently lacking evidence. The purpose of this study is to compare the efficacy and safety of EA with sham acupuncture (SA) in the treatment of OIC in cancer patients.

2.3 Study Hypothesis

We hypothesize that EA is better than SA in treating OIC in adult patients with cancer pain.

2.4 Methodology

2.4.1 Trial Design

This is a multicenter, prospective, sham-controlled, parallel-group, subject- and assessor-blinded, randomized trial at 7 centers in China. Cancer patients must meet the Rome IV[10] diagnostic criteria for OIC.

2.4.1.1 Randomization

Web-based central randomization will be performed by the Linkermed Pharm Technology Co. Ltd (Beijing, China). Participants will be randomly allocated, in a 1:1 ratio, to either the EA or the SA group using permuted block-randomization. Acupuncturists in each center will be responsible for getting random numbers. Via inputting the screening information of the participant in the central randomization system through the web, they will get the random number and group allocation.

2.4.1.2 Blinding

In this study, participants, outcome evaluators, and data analysts will be blinded to the group assignments. The acupuncturists who perform the treatment will not be blinded due to the nature of the acupuncture treatment. Participant blinding will be achieved via a minimal needling at non-acupoints. Bilateral sham points will be attached with the same EA apparatus using a continuous wave of 10Hz and a current intensity of 0.1–0.2mA for 30 minutes after a brief activation period of 30 seconds.

For blinding assessment, all participants will be requested to answer the following question: “Is EA the acupuncture modality that you have received?”within five minutes after any treatment at week 8.

2.4.1.3 Sample Size

On the basis of unpublished data, a 14% response rate was assumed for the sham acupuncture group in this study. We estimated that a sample size of 100 participants would provide 90% power to detect a between-group difference of 31.4% at the two-sided significance level of 0.05 and 15% loss to follow-up.

2.4.2 Subjects

Participants with cancer will be publicly recruited from inpatient and outpatient departments through posters and networks from 6 centers in China.

2.4.2.1 Inclusion Criteria

(1) Cancer patients must meet the Rome IV[10] diagnostic criteria for OIC. Participants have at least 2 of the following new or worsening symptoms of constipation following initiation, alteration, or increase in opioid treatment: fewer than three SBMs per week, straining (>25% of defecations), sensation of incomplete evacuation (>25% of defecations), lumpy or hard stools (>25% of defecations), and/or sensation of anorectal obstruction/blockage (>25% of defecations). For patients with a history of chronic functional constipation, he/she must have worsening symptoms of constipation when the opioid therapy is initiated, changed, or the dose is increased;(2)Patients recruited in this trial must have a history of OIC symptoms for at least 1 week;(3) Patients must be $\geq$18 years of age and $\leq$85 years of age; (4) Patient's cancer condition must be stable with a life expectancy that is more than six months; (5)Patients must have an Eastern Cooperative Oncology Group (ECOG)[27]performance status of 0-3; (6)Patients must have been receiving a stably maintained opioid regimen, consisting of a total daily dose of 30 mg to 1000 mg oral morphine equivalents for at least 2 weeks prior to screening for cancer pain. Furthermore, it must be anticipated that the opioid will be maintained for at least 10 weeks;(7)The SBM frequency of the patients must be $\leq$ 2 times a week when laxatives are not being taken;(8)Patients must be capable of oral intake of drugs, food and beverages; (9)Provision of written informed consent before inclusion.

2.4.2.2 Exclusive Criteria

Participants will be excluded from this trial if they have any of the following conditions:

(1) Patients diagnosed with clinically significant abnormal defecation due to functional

disorders or structural abnormalities of the gastrointestinal tract and other tissues related to gastrointestinal tract (not including OIC): inflammatory bowel disease, irritable bowel syndrome, rectal prolapse, gastrointestinal obstruction, peritoneal metastasis, or peritoneal tumor at the time of enrollment; (2)Patients with a history of gastrointestinal tract operation, abdominal operation, or abdominal adhesion within one month prior to screening; history of intestinal obstruction within three months prior to screening; (3)Diagnosis of active diverticular disease; or severe hemorrhoid; or anal fissure; or artificial rectum or anus;

(4) Patients with an intraperitoneal catheter or those that use a feeding tube to maintain vital signs; (5)Diagnosis of pelvic disorder, which are considered to have obvious effects on the intestinal transport of feces (such as uterine prolapse ≥degree 2, uterine fibroids [located in the posterior of the uterus with a diameter ≥ 5 cm] affecting bowel movement); (6)Patients that are being treated with a new cancer chemotherapy, which had never been administered in the past, within 14 days of the screening or are scheduled to receive such therapy during the study; (7)Patients that received radiotherapy within 28 days of the screening or are scheduled to receive such therapy during the study; (8)Patients that underwent a surgery or intervention that is considered to have an obvious effect on the gastrointestinal functions within 28 days of the screening or are scheduled to receive surgery or intervention which is considered to have obvious effects on the gastrointestinal functions during the study, or scheduled to receive surgery or intervention which will be anticipated to prevent the patients from completing the trial; (9)Patients with uncontrolled hyperthyroidism, severe hypertension, heart disease, systematic infection or blood coagulation disorders (hypercoagulation status or hemorrhagic tendency); (10)Patients that consumed >4 additional opioid doses per day, for breakthrough pain, for more than 3 days during the baseline period, or if their maintenance opioid dosing regimen was modified during this period; (11)Patients with severe cancerous pain (e.g., typical average daily pain intensity rating of 7 to 10 on a numerical rating scales (NRS; 0 [no pain] to 10 [the worst pain possible]) after the utility of routine dose and frequency of opioids) refractory to opioid therapy;(12) Patients with a history of opioid discontinuation due to severe adverse events or patients that are suspected to discontinue opioid use due to the potential risk of adverse events; (13)Patients that received an opioid receptor antagonist

or agonist within one month of the screening, or those who are scheduled to receive such therapy during the study; (14) Patients with a history of nerve neurolysis;(15) Patients with severe cognitive impairment, aphasia, or psychiatric disorders; abdominal aortic aneurysm; hepatomegaly(liver span > 14cm at the mid-clavicular line by ultrasound examination); or splenomegaly (spleen length [cranial to caudal] > 13cm by ultrasound examination);(16) Patients that have received acupuncture within three months of the screening; (17)Other patients who are considered ineligible for the study by the investigator on the basis of concomitant therapy and medical findings.

2.4.2.3 Subject Withdrawals

There will be at least one oncologist or gastroenterologist in each center. They will assess the severe adverse events (SAEs) and then determine whether the participant to continue or terminate the trial. Subjects may leave the study at their own discretion, or the investigator may determine whether it is in the best interest of subjects to withdraw from the trial due to worsening of symptoms, or the occurrence of a serious adverse event.

2.4.2.4 Subject Recruitment, Screen and Grouping Assignment

Participants with cancer will be publicly recruited from inpatient and outpatient departments through posters and networks. Research assistants of each site will preliminarily screen the participants by recording their disease condition, history of the disease and treatment, and the demographic data. An oncologist or gastroenterologist of each site will take charge of the diagnosis and the differential diagnosis of the OIC. Potential participants will fill out a 1-week patient diary to record bowel movements, the stool consistency, degree of difficulty in defecation, the rescue medicine drugs and duration of usage, and the intensity of cancerous pain, etc. Eligible participants then will be randomized to EA or SA group. Acupuncturists are in charge of the participants' group assignment, and the EA or SA treatments. They are also responsible for the assessment of safety during treatment. During the trial, the professional evaluators of each site will instruct the participants how to fill in patients' self- assessment related to the trial and their patient diaries and the evaluators will record the data on the case report form (CRF) through the whole trial period. The subject flow was shown in Figure 1.

Figure 1. Subject flow

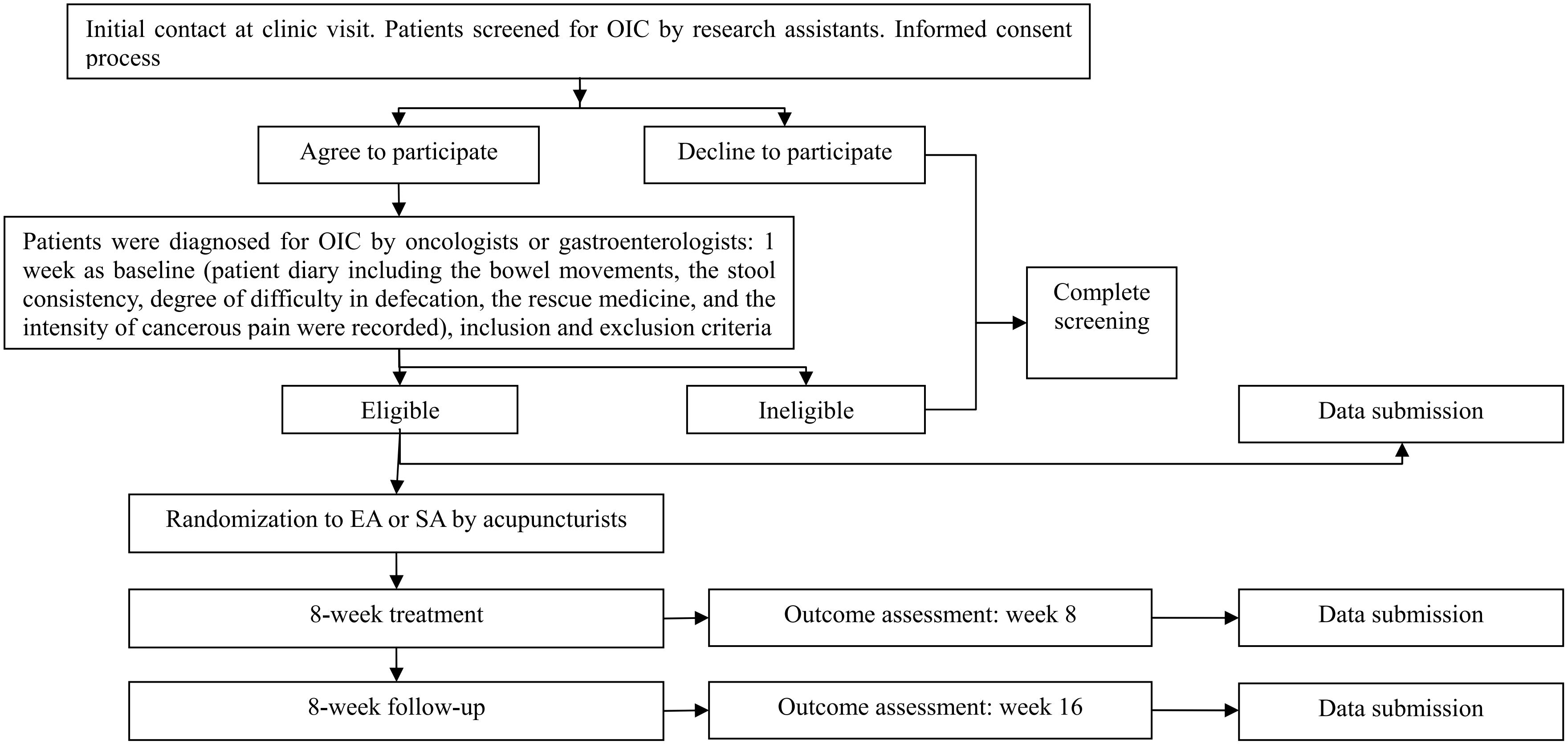

2.4.3 Trial flow chart

Figure 2.Trial flow chart

Enrollment	Study Period			
	**Baseline**	**Allocation**	**Treatment**	**Follow-up**
	Weeks -1	Week 0	Weeks 1 to 8	Weeks 13 to 16
Eligibility criteria	×			
Demography characteristics	×			
Disease history of cancer	×			
Disease history of OIC and constipation	×			
Eligibility screen	×			
Informed consent	×			
Allocation		×		
**Interventions**				
Electroacupuncture			×	
Sham electroacupuncture			×	
**Assessments**				
SBMs	×		×	×
CSBMs	×		×	×

Mean Bristol Stool Form Scale score for stool consistency of SBM	×		×	×
Mean score for straining of SBM	×		×	×
PAC-SYM total score and subscale scores	×		×	×
PAC-QOL total score and subscale scores	×		×	×
Patients' global assessment of treatment efficacy			×	×
Rescue medicine usage	×		×	× (weeks 9-16)
Opioid usage	×		×	× (weeks 9-16)
Patients' expectation of the acupuncture efficacy	×			
Blinding assessment			×	
Cancer pain			×	×
Adverse events	×		×	× (weeks 9-16)
Safety assessment	×		×	× (weeks 9-16)

Abbreviations: OIC, Opioid-induced constipation; SBMs, spontaneous bowel movements; CSBMs, complete spontaneous bowel movements; PAC-SYM, Patient Assessment of Constipation-Symptom questionnaires; PAC-QOL, Patient Assessment of Constipation-Quality of Life questionnaires.

Figure 3. The schedule of enrollment, interventions, and assessments

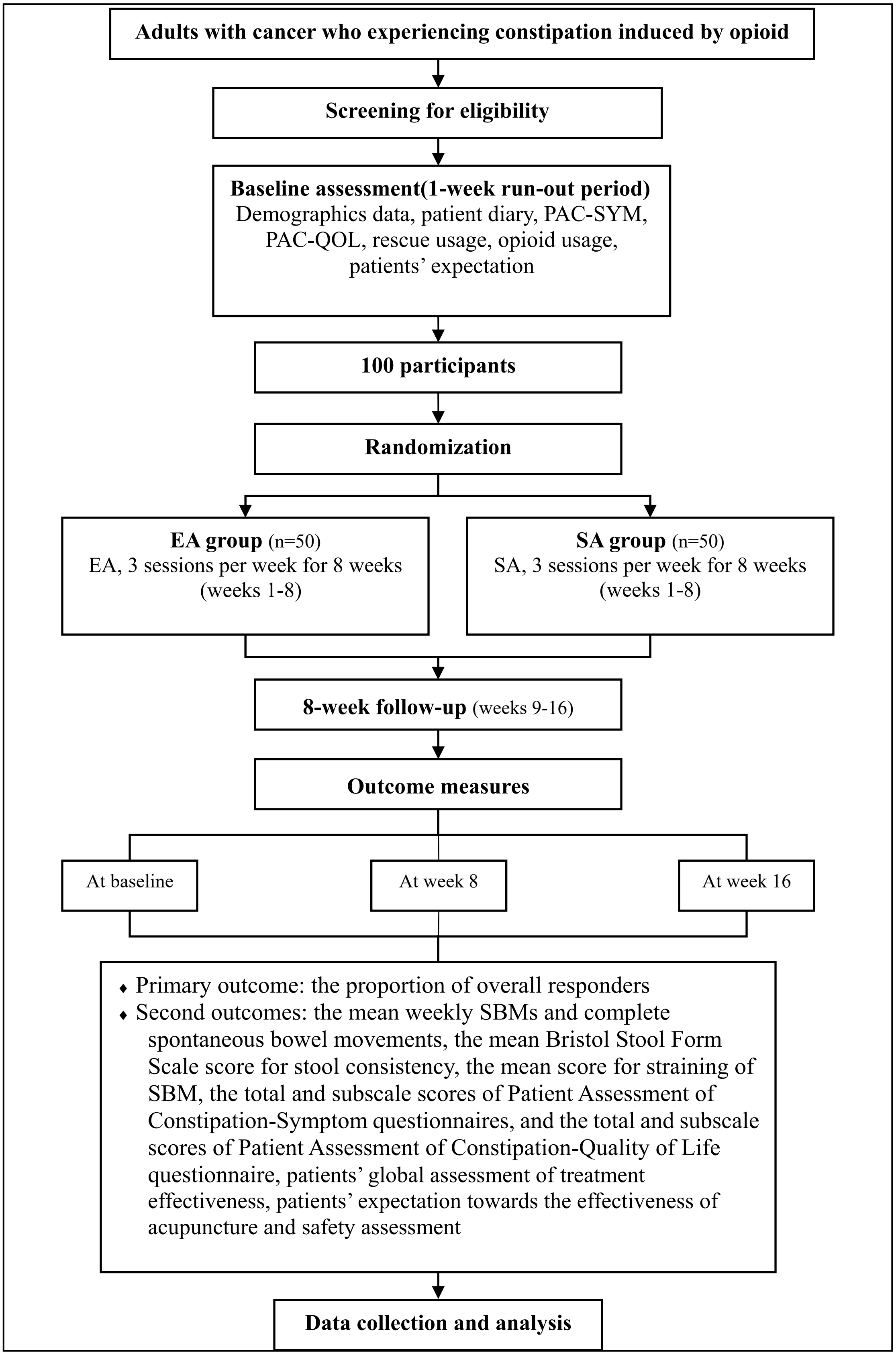

2.4.4 Outcomes Measurement

2.4.4.1 Primary Outcome

The primary outcome will be the proportion of overall responders, defined as a patient that has ≥3 SBMs/wk and ≥ increase of 1 SBM from baseline simultaneously for at least 6 out of 8 weeks of the treatment period. SBM refers to a bowel movement that occurred without medication or assistance within the previous 24 hours. When a bowel movement occurs within 24 hours of the use of an optional assisted method (rescue medication or other bowel-treatment regimens) for defecation, it is not regarded as an SBM.

Every participant will be required to keep a diary 13 weeks: baseline (run-out period before randomization), 8 weeks of treatment, and 4 weeks of follow-up. Diary entries include the frequency of bowel movements, the consistency of the stool, the difficulty in defecating, the rescue medicine drugs applied and their duration, and the intensity of the cancer pain. During the treatment period, the diary will be collected weekly, and during the follow-up period, it will be collected at the end of week 16. The outcome evaluators will examine the diary content and determine the SBM and frequency accordingly.

2.4.4.2 Secondary Outcomes

(1) Changes in the mean weekly SBMs from the baseline during weeks 1-8 and weeks 13-16. The mean weekly SBMs equals the total frequency of SBMs divided by the numbers of week(s) recorded. Assessment time frame: at baseline, over weeks 1-8 and 13-16.

(2) The proportion of patients with ≥3 mean weekly SBMs during weeks 1-8 and weeks 13-16. Assessment time frame: at baseline, over weeks 1-8 and 13-16.

(3) The proportion of patients with an increase of ≥1 mean weekly SBM from the baseline during weeks 1-8 and weeks 13-16. Assessment time frame: at baseline, over weeks 1-8 and 13-16.

(4) A change in the mean weekly CSBMs from the baseline during weeks 1-8 and weeks 13-16. A CSBM is defined as an SBM with the feeling of complete evacuation. The mean weekly CSBMs equals the total frequency of CSBMs divided by number of week(s)

recorded. Assessment time frame: at baseline, over weeks 1-8 and 13-16.

(5) The proportion of patients with ≥3 mean weekly CSBMs during weeks 1-8 and weeks 13-16. Assessment time frame: at baseline, over weeks 1-8 and 13-16.

(6) The proportion of patients with an increase of ≥1 mean weekly CSBM from the baseline during weeks 1-8 and weeks 13-16. Assessment time frame: at baseline, over weeks 1-8 and 13-16.

(7) A change in the mean Bristol Stool Form Scale score for stool consistency of SBMs from the baseline during weeks 1-8 and weeks 13-16. For stool consistency, each patient will be asked to record their stool consistency according to the Bristol Stool Form Scale[28] on the following seven points scale (scored from 1 to 7 for stool types 1 to 7, respectively). Assessment time frame: at baseline, over weeks 1-8 and 13-16.

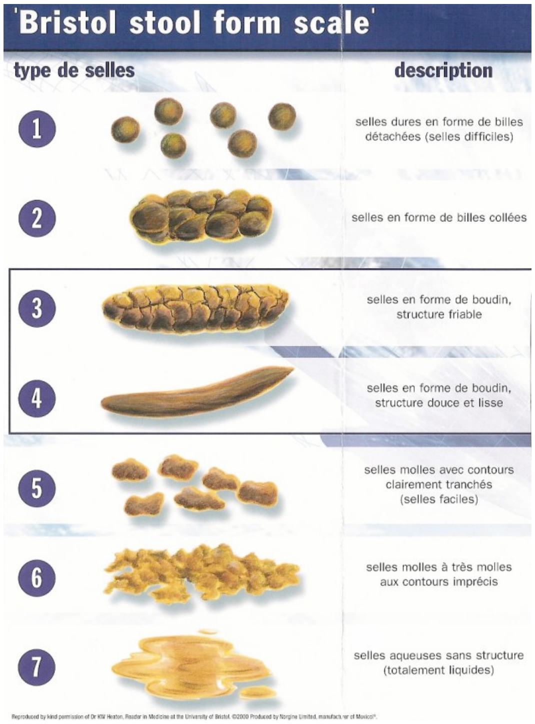

- Type 1: Separate hard lumps, like nuts (hard to pass)
- Type 2: Sausage-shaped, but lumpy
- Type 3: Like a sausage but with cracks on its surface
- Type 4: Like a sausage or snake, smooth and soft
- Type 5: Soft blobs with clear cut edges (passed easily)
- Type 6: Fluffy pieces with ragged edges, a mushy stool
- Type 7: Watery, no solid pieces. Entirely liquid

(8) A change in the mean score for the straining of SBMs from the baseline during weeks 1-8 and weeks 13-16. For assessment of the straining of SBMs, each patient will be asked to rate his/her score of straining, using the following five-point scale[29]: not at all difficult (0), a little bit difficult (1), moderately difficult (2), quite a bit difficult (3), extremely difficult (4). Assessment time frame: baseline, over weeks 1-8 and 13-16.

(9)A change in the total and subscale score of the Patient Assessment of Constipation-Symptom (PAC-SYM) questionnaire from the baseline at weeks 8 and 16. The PAC-SYM is a questionnaire used to evaluate the severity of chronic constipation in the past 2 weeks. It consists of 12 items, which are subdivided into abdominal (4 items), rectal (3 items), and stool (5 items) scales.34 36 The score of each item ranges from 0 to 4, with 0 = symptom absent, 1 = mild, 2 = moderate, 3 = severe and 4 = very severe. Lower scores indicate a lower symptom burden. Each subscale score will be calculated as the mean of the completed items for that subscale. The total score will be calculated as the mean of all completed items. In this trial, the Chinese version of PAC-SYM, which has been validated to have a satisfactory psychometric property[30], will be used. Assessment time frame: at baseline, at weeks 8 and 16.

(10) A change in the total and subscale scores of the Patient Assessment of Constipation-Quality of Life (PAC-QOL) questionnaires from the baseline at weeks 8 and 16. The PAC-QOL is a 28-item self-reported questionnaire to assess the burden of constipation on patients' everyday functioning and well-being in the 2 weeks (14 days) prior to assessment[31]. This questionnaire is divided into four subscales: physical discomfort (items 1-4), psychosocial discomfort (items 5-12), worries/concerns (items 13-23), and satisfaction (items 24 to 28). Each of the item scores ranges from 0 (not at all) to 4 (extreme), with lower scores indicating a better quality of life. For each visit, individual subscale scores will be calculated as the mean of the completed items for that subscale. The total score will be calculated as the mean of all of the completed items. We will use the Chinese version of this test[32] in our trial, which has been demonstrated to be a reliable and valid tool. Assessment time frame: at baseline, at weeks 8 and 16.

(11) Patients' global assessment of treatment efficacy. Each patient will be asked to rate his/her efficacy of treatment using the following 7-point self-reporting scale: markedly worse (1), moderately worse (2), slightly worse (3), no change (4), slightly improved (5), moderately improved (6), markedly improved (7). Scales with seven response categories are easy to use and have shown a high reliability and validity[33]. This questionnaire will be completed at week 8 and week 16. Assessment time frame: baseline, at weeks 8 and 16.

(12) The proportion of patients using rescue medicine and the mean frequency of rescue medicine use per week during weeks 1-8 and weeks 9-16. Assessment time frame: at

baseline, over weeks 1-8 and 13-16.

Other Pre-specified Outcome Measures

(13) The proportion of patients discontinuing the opioid, and those with a ≥30% weekly mean increase or decrease in the dose of opioid from baseline during weeks 1-8 and weeks 9-16. Assessment time frame: at baseline, at weeks 8 and 16.

(14) The proportion of patients with a change from baseline in anti-tumor therapy that could impair the defecation during weeks 1-8 and weeks 9-16. Assessment time frame: at baseline, at weeks 8 and 16.

(15) Patients' belief in the efficacy of acupuncture. Participants will be asked to answer the following questions at baseline: "Do you think acupuncture will be effective in treating the disease in general?" and "Do you think acupuncture will be effective in improving the OIC?" For each question, patients will choose one of the following answers: "unclear/whatever", "Yes", or "No". Assessment time frame: at baseline.

(16) Blinding assessment. The blinding is regarded as successful when a patient guesses he/she has received a conventional EA. Before treatment, we told patients that they had a 50% chance of receiving conventional electroacupuncture (EA) with a deeper insertion versus minimal electroacupuncture (SA) a superficial penetration. Conventional electroacupuncture and minimal electroacupuncture have a possible similar efficacy. Both treatments used a relatively small electric intensity, and they may or may not feel the stimulation during treatment. Patients were treated separately to avoid communication. To assess the success of blinding, within 5 minutes after treatment at week 8, patients were asked to guess whether they received conventional EA. Assessment time frame: at week 8.

3. Safety Assessment

All adverse events (AEs) will be recorded throughout the whole trial in Adverse Event Form (AEF) by patients themselves and outcome assessors. In our trial, the serious AEs will be defined as events that cause death, exacerbation of the preexisting condition, interruption of treatment, prolongation of existing hospitalization, permanent disability or

damage, or required medical intervention to prevent one of the above outcomes. AEs will be categorized as treatment related or non-treatment related based on its potential association with acupuncture needling procedure by acupuncturists and related specialists within 24 hours. The treatment related AEs defined as follows: dizziness, fainting, localized hematoma, localized minor infection, or some discomforts after acupuncture. Safety assessments also include an 11-point NRS (0 indicates no pain, and 10 indicates the severest pain) to evaluate the intensity of cancer pain. The mean and largest intensity of cancer pain during the preceding week will be evaluated at baseline, as well as weeks 2, 4, 6, 8 and 16.

4. Interventions

The intervention scheme of this trial is based on our previous trials regarding acupuncture for functional constipation[24, 25]. Acupuncturists who had an acupuncture license and at least 2 years of clinical experience in acupuncture will perform the treatment. We will use disposable acupuncture needles (of the following sizes: 0.30 × 40, 0.30 × 50 and 0.30 × 75 mm) and SDZ-V EA apparatus (all Hwato Brand, Suzhou Medical Appliance Factory, Suzhou, China) in this trial. The duration of the trial for each participant will be 17 weeks: 1- week baseline assessment (run-out period), 8- week treatment and 8- week follow- up.

4.1 EA

Bilateral Tianshu (ST25), Fujie (SP14), Shangjuxu (ST37) will be used in the EA group. The location of the acupoints will be based on Nomenclature and location of acupuncture points[34] drafted in 2006 by the National Standard of the People's Republic of China (GB/T 12346–2006). The local skin will be routinely sterilized while the patient is in a supine position. For ST25 and SP14, 0.30×50 mm or 0.30×75 mm needles will be gently vertically inserted to the muscle layer of the abdominal wall, where patients will feel sharp pain and acupuncturists will feel resistance from the needle tip. For ST37, 0.30×40 mm needles will be vertically inserted approximately 15 mm deep, followed by three-time manipulation of even lifting and twisting method to elicit the sensation of deqi. Paired alligator clips of the EA apparatus will then be attached to the needle holders of

the bilateral ST25, SP14, and ST37. The stimulation will be retained for 30 minutes, with a continuous wave of 10 Hz and current intensity of 0.5 to 4 mA. All needles will be removed after 30 minutes and pressure will be applied using a dry sterilized cotton ball to avoid bleeding. Patients will be followed up for another 8 weeks after the treatment stopped.

4.2 SA

The patients in the SA group will receive minimal needling at non-acupoints as bilateral sham ST25, SP14, and ST37. The sham ST25 and SP14 are located 2 cm horizontally outward of the points stimulaed in the EA group. The sham ST37 point is located outward of ST37 in the middle of the stomach and gallbladder channel. After sterilization of the skin, 0.30×40 mm needles will be directly inserted about 2-3 mm until they can stand up when attached by the alligator clips. No manipulation will be used and no deqi sensation will be elicited at any of the sham points. The bilateral sham ST25, SP14, and ST37 points will be attached by the same EA apparatus with a continuous wave of 10 Hz and current intensity of 0.1 to 0.2 mA for 30 minutes with only the initial 30 seconds on.

Patients in both groups will receive 24 treatment sessions over an 8-week period (3 sessions each week, ideally every other day). Each session will last for 30 minutes. Patients will be treated separately to prevent between-patient communication. Patients will be followed up for another 8 weeks after the treatment stopped.

4.3 Rescue medication

During the trial, other medication or intervention for OIC will be discouraged. However, if a patient has no bowel movement for 72 consecutive hours and cannot tolerate it, only bisacodyl (5 to 10 mg; up to 20 mg per day) or a 110ml glycerol enema will be permitted as a rescue medication. Details of drug use (time and frequency) will be recorded.

5. Informed consent

Informed Consent: Study Introduction

Dear participants：

Opioid analgesics, such as morphine and oxycodone, are recommended as the cornerstone for the management of moderate to severe cancer pain by WHO Cancer Pain Relief Guidelines. Opioid-induced constipation (OIC) is the most prevalent serious adverse events (AEs). It is reported in 94% of cancer patients who take opioids for pain. OIC is defined as a change in baseline bowel habits or defecatory patterns following the initial administration or modification of opioid therapy.

Unlike many other opioid-related AEs, the symptoms of OIC tend to be persistent and difficult to tolerate, which can adversely reduce patients' quality of life. The mechanism of OIC involves multiple contributing factors: exogenous opioids can activate μ-receptors throughout the gastrointestinal tract and lead to a change in gut motility, a decrease in gut secretion and an increase in sphincter tone, which will result in OIC. The management of OIC is multifaceted, involving a combination of pharmacological and non-pharmacological interventions, such as laxatives and increased fluid and fiber intake. However, the efficacy of these interventions is limited and these approaches do not address all of the underlying pathophysiological mechanisms of OIC. Recently, peripherally acting μ-opioid receptor antagonists (PAMORAs), such as naloxegol and methylnaltrexone, have been shown to be effective in treating OIC patients who response poorly to simple laxatives. However, these drugs are still under test in clinical trials with unclear long-term efficacy and safety; they have not been approved for use in China. In addition, the use of PAMORAs is often accompanied by AEs of abdominal pain and flatulence. At present, traditional Chinese medicine, glycerin enema and other methods are also can be used to treat OIC.

If you have been experiencing cancer-related pain and were haunted by the symptom of OIC. We invite you to participate in the study. This study was supported and funded by the 2019 National Administration of Traditional Chinese Medicine "Project of building evidence-based practice capacity for TCM--Project BEBPC-TCM" (NO. 2019XZZX-ZJ). The objective of this study is to assess the efficacy and safety of EA compared to sham

acupuncture (SA) in the treatment of OIC in patients with cancer. Participating in this study can relieve symptoms of OIC while also contributing to the development of medicine, especially for acupuncture and moxibustion of Traditional Chinese medicine.
Patients with the following conditions should not participate in this study: If you cannot participate in the study, we will provide free scale testing and related consultation.

(1) Patients diagnosed with clinically significant abnormal defecation due tostructural abnormalities of the gastrointestinal tract and other tissues related to gastrointestinal tract (not including OIC): inflammatory bowel disease, rectal prolapse, gastrointestinal obstruction, peritoneal metastasis, or peritoneal tumor at the time of enrollment;
(2) Patients with a history of gastrointestinal tract operation, abdominal operation, or abdominal adhesion within one month prior to screening; history of intestinal obstruction within three months prior to screening;
(3) Diagnosis of active diverticular disease; or severe hemorrhoid; or anal fissure; or artificial rectum or anus;
(4) Patients with an intraperitoneal catheter or those that use a feeding tube to maintain vital signs;
(5) Diagnosis of pelvic disorder, which are considered to have obvious effects on the intestinal transport of feces (such as uterine prolapse ⩾degree 2, uterine fibroids [located in the posterior of the uterus with a diameter ⩾ 5 cm] affecting bowel movement);
(6) Patients that are being treated with a new cancer chemotherapy, which had never been administered in the past, within 14 days of the screening or are scheduled to receive such therapy during the study;
(7) Patients that received radiotherapy within 28 days of the screening or are scheduled to receive such therapy during the study;
(8) Patients that underwent a surgery or intervention that is considered to have an obvious effect on the gastrointestinal functions within 28 days of the screening or are scheduled to receive surgery or intervention which is considered to have obvious effects on the gastrointestinal functions during the study, or scheduled to receive surgery or intervention which will be anticipated to prevent the patients from completing the trial;

(9) Patients with uncontrolled hyperthyroidism, severe hypertension, heart disease, systematic infection or blood coagulation disorders (hypercoagulation status or hemorrhagic tendency);

(10) Patients that consumed >4 additional opioid doses per day, for breakthrough pain, for more than 3 days during the baseline period, or if their maintenance opioid dosing regimen was modified during this period;

(11) Patients with severe cancerous pain (e.g., typical average daily pain intensity rating of 7 to 10 on a numerical rating scales (NRS; 0 [no pain] to 10 [the worst pain possible]) after the utility of routine dose and frequency of opioids) refractory to opioid therapy;

(12) Patients with a history of opioid discontinuation due to severe adverse events or patients that are suspected to discontinue opioid use due to the potential risk of adverse events;

(13) Patients that received an opioid receptor antagonist within one month of the screening, or those who are scheduled to receive such therapy during the study;

(14) Patients with a history of nerve neurolysis;

(15) Patients with severe cognitive impairment, aphasia, or psychiatric disorders; abdominal aortic aneurysm; hepatomegaly(liver span > 14cm at the mid-clavicular line by ultrasound examination); or splenomegaly(spleen length [cranial to caudal] > 13cm by ultrasound examination);

(16) Patients that have received acupuncture within three months of the screening;

(17) Other patients who are considered ineligible for the study by the investigator on the basis of concomitant therapy and medical findings.

We plan to enroll a total of 100 participants with 50 in each group in this trial. If the patients can participate in this study, doctors will randomly assign them to conventional electroacupuncture(EA) group or minimal electroacupuncture group. Each patient will have a 50% chance to be in the EA group or minimal electroacupuncture group. Patients in both groups will receive 24 treatment sessions over an 8-week period (3 sessions each week, ideally every other day). Patients will be followed up for another 8 weeks after the treatment stopped. During the study period, subjects are required to cooperate with doctors to complete relevant scales and carry out necessary auxiliary examinations, as well as to adherence to the schedule for treatment, examination, and

follow-up visit. Additionally, you are also responsible for reporting any changes in your physical and mental status to your doctor during the study process regardless of whether you think these changes are related to the study or not. During the trial, other medication or intervention for OIC will be discouraged. However, if a patient has no bowel movement for 72 consecutive hours, only bisacodyl (5 to 10 mg; up to 20 mg per day) or a 110ml glycerol enema will be permitted as a rescue medication. Details of drug use (time and frequency) will be recorded. The doctors will make every effort to prevent and treat any side effects brought on by this study. During acupuncture treatment, you may feel soreness, numbness, heavy, distension sensation, etc., which are normal reactions to acupuncture. Acupuncture treatment may have some adverse effects (e.g., dizziness, fainting, localized hematoma, localized minor infection), but it is rare and mild. We promise that in case of adverse events, we will do our best to provide treatment in accordance with the routine diagnosis and treatment according to professional judgment, and will follow the condition until it stabilizes or until the event is otherwise explained. The hospital will bear all the costs. Free treatment, consultation and scale measurement will be provided throughout the trial (including the follow-up period). You and your legal representative will be promptly notified of any information that may affect the subject's participation in the study. Whether to participate in this study will be entirely determined by the patients themselves, and the subjects' privacy will be kept strictly confidential within the scope of the law. Only the institutes responsible for the study, clinical research institutes, and ethics committees may have access to your medical records to verify clinical trial procedures and data. Your name will not appear in any publications or reports related to this study. Subjects have the right to withdraw from the study at any time during the study without any discrimination or retaliation, and without affecting any medical services. For your best interests(such as unbearable acupuncture pain or severe AEs), researchers may terminate your participation at any time during the study. Personal data of participants in the study are kept confidential. If you need more information, feel free to talk to your doctor. Subjects are entitled to ask our physicians at any time and to contact the ETHICS committee office if they have complaints.

Patient statement

I have been informed of the research purpose, content and method of this research, and I have fully understood the nature, significance, and possible risks and benefits of this research. I have the right to participate in this study voluntarily or not.My personal information will be kept confidential.I grant access to the study data to the DRUG regulatory agency or the ethics committee.

I have fully understood the above and made the decision on my own after full consideration: I volunteered to be a subject in the study "Effects of electroacupuncture on opioid-induced constipation in patients with cancer: a multicenter randomized controlled trial". I am willing to accept research requests and cooperate with researchers.I am willing to actively cooperate with relevant examinations and fulfill the rights and obligations of subjects to ensure the final completion of this study.

Signature of patient Year month day

Telephone:

Researcher declaration:

I have carefully explained to the subjects the situation of this study and the benefits and risks of participating in this study.His signature is valid.Medical problems, language or education do not preclude an understanding of the above.

Signature of researcher Year month day

Telephone:

:

6. Quality Control

All staff members will undergo training prior to the trial. Monitors will check the case report forms and the acupuncture operation regularly. To improve adherence to intervention protocols, the majority of patients will come from the inpatient setting. The outcomes will be evaluated by independent assessors who are unaware of the group allocation. The data will be input by a clinical research coordinator according to the

contents of CRF using the Electronic Data Capture System (EDC), which will be monitored by Clinical Research Associate. Detailed documentation of drop-outs and withdrawals, including the reasons, will be obtained throughout the trial. All of the investigators will always maintain a strict privacy policy to protect confidentiality before, during and after the trial.

7. Data Management

7.1 The Raw Data Management and Archiving

We use Electronic Data Capture System (EDC) system to perform data entry. The research assistants will fill out all the electrical CRF through RDC system. Researchers will inspect the eCRF, and signed electrically for the eCRF going into effect. The eCRF and the trace of eCRF revising will be left in the Oracle database.

7.2 Data Entry and Storage

7.2.1 Database Building and Testing, Data Entry Interface

The eCRF will be noted through CDISC SDTM standard, and the data entry interface will be generated through the Oracle Clinical software. The data entry interface should be in accordance with the paper-version CRF as far as possible. The inputted data will be stored in the Oracle database.

After preliminarily setting up the database, the entry clerks will input some analog data according to the CRF to test the database. The testing contains: (1) the agreement of the data entry interface and the paper-version CRF; (2) the agreement of the exported data from the database and the analog data; (3) the agreement of the structure of the exported database and the paper-version CRF. After the testing, data administrators should revise the database and make a testing report. Then they electrically signed on the approval page of the database to indicate that the testing is completed. The electrical files of the analog

CRF, Noted CRF, screenshot of the data entry interface, database testing report, and the approval page of the database should be saved. If the database updates during the trial, the electrical files mentioned above are also need to be updated.

7.2.2 Data Entry and Inspection

The research assistants take charge of the data entry for our trial. Before the entry, all the research assistants will accept the related training according to the data entry handbook. Researchers will inspect the database, and then sign electrically to let the data go in to effect.

7.3 Data Verification and Problems Solving

Researchers will verify the data through Data Verification Plan (DVP) approved by the data administrator and the statisticians. Data queries will be inputted to a data query database, and form the DCF. After being inspected, the DCF will then be handed back to the original site, and the researchers of the site should answer the queries. Any revision of the database will be recorded through the RDC software.

7.4 Medical Coding

A data administrator who has the medicine background will take charge of the medical coding. The contents of the coding are the clinical history, adverse events, and combined medication. The clinical history and adverse events will be coded through MedDRA dictionary (Version 13.0), and the combined medication will be coded via WHO DD dictionary (Version 2007.03). The lead researchers will verify the coded e-files.

7.5 Data Report

Data report contains the aspects as followed: (1) members of the project; (2) disagreement from the primary data management plan; (3) actual finish time of every project; (4) problems and the solution during the data management (if have any); (5) reconstruction of the database (if have any); (6) distribution of the participants; (7) participants who disobey the trial protocol; (7) classifying plan of the statistical analysis population.

Data report will be performed monthly since the first entry of the eCRF.

7.6 Data Auditing

When the data checking is finished, a data auditing and blinding review meeting will be hold. On the meeting, the data administrators, statisticians, researchers, clinical inspectors, and other related members would have a discussion on the following items according to the data management report and the data lists:

- Distribution of the participants;
- Protocol disobeying or not;
- Possible outlier;
- Baseline data;
- Outcomes;
- Statistical analysis plan.

7.7 Database Locking

The database will be locked if it fulfills all the aspects as followed: All the queries have been solved, and the database has been updated; No query has been found through the data inspection; The medical coding has been completed; The plan of the participants ' classification has been approved; The final draft of the SAP has been made, and approved by the project leader.

The statisticians and the data administrators will signed the data locking form, and then the database will be locked. The locked database will be sent to the statisticians for further statistical analysis through the data format of SAS.

8. Statistical Consideration

The following is an overview of the statistical considerations. Details of the pre-specified statistical analyses can be found in the Statistical Analysis Plan (SAP).

8.1 Statistical Analysis

The primary study hypothesis is that EA is more effective than SA in the treatment of OIC in patients with cancer. The primary outcome is the proportion of overall responders, defined as a patient that has ≥ 3 SBMs/wk and ≥ increase of 1 SBM from baseline simultaneously for at least 6 out of 8 weeks of the treatment period. The primary analysis will be intention-to-treat, which is defined as all randomized participants.
The following secondary outcomes will be analyzed using the t test, repeated measures analysis, Wilcoxon rank-sum test, Chi-square test or Fisher's exact test, as appropriate:
A two-side test with p<0.05 will be considered significant for all analyses.

8.2 Statistical Analysis Plan (SAP)

Prior to database lock and before code breaking, a final version of the SAP shall be issued and approved by the study statistician, and the principal investigator. The SAP will define all "pre-specified, planned analyses" and provide the general specifications for the analysis of the data to be collected and presented in the Clinical Study Report.

9 Ethical principle

For every study site, only when the trial protocol is approved by the IRB, the enrollment of participant will begin, but all should be after May 1, 2019.

10 Funding

This study was supported and funded by the 2019 National Administration of Traditional Chinese Medicine "Project of building evidence-based practice capacity for TCM--Project BEBPC-TCM" (NO. 2019XZZX-ZJ) by China Academy of Chinese Medical Sciences.

11 References

1. Caraceni A, Hanks G, Kaasa S, et al: Use of opioid analgesics in the treatment of cancer pain: Evidence-based recommendations from the EAPC. *Lancet Oncol* 2012;13(2):e58-68.
2. Mercadante S. Intravenous morphine for management of cancer pain. *Lancet Oncol* 2010;11(5):484-9.
3. World Health Organization. Cancer Pain Relief With A Guide To Opioid Availability. 2nd ed. Geneva, Switzerland: World Health Organization; 1996.
4. Cherny NI. The pharmacological management of cancer pain. *Eur J Cancer* 2001;37 Suppl 7:S265-78.
5. Luo M, Li L, Lu CZ, *et al.* Clinical efficacy and safety of lactulose for minimal hepatic encephalopathy: a meta-analysis. *Eur J Gastroenterol Hepatol* 2011; 23: 1250–7.
6. Kessler ER, Shah M, Gruschkus SK, *et al.* Cost and quality implications of opioid-based postsurgical pain control using administrative claims data from a large health system: opioid- related adverse events and their impact on clinical and economic outcomes. *Pharmacotherapy* 2013; 33(4):383–91.
7. Oderda GM, Said Q, Evans RS, *et al*. Opioid-related adverse drug events in surgical hospitalizations: impact on costs and length of stay. *Ann Pharmacother* 2007;41(3):400–407.
8. Poulsen JL, Brock C,Olesen AE, *et al*. Evolving paradigms in the treatment of opioid-induced bowel dysfunction. *Therap Adv Gastroenterol* 2015; 8(6):360-72.
9. Bell TJ, Panchal SJ, Miaskowski C, *et al*. The prevalence, severity, and impact of opioid-induced bowel dysfunction: results of a US and European Patient Survey (PROBE 1). *Pain Med* 2009;10(1):35-42.
10. Mearin F, Lacy BE, Chang L, *et al*. Bowel disorders. *Gastroenterology* 2016; 150(6): 1393–407.
11. Kalso E, Edwards JE, Moore RA, *et al*. Opioids in chronic non-cancer pain: systematic review of efficacy and safety. *Pain* 2004;112(3):372–80.
12. Sykes NP. The relationship between opioid use and laxative use in terminally ill

cancer patients. *Palliat Med* 1998;12(5):375–82.

13. Morlion B, Clemens KE, Dunlop W. Quality of life and healthcare resource in patients receiving opioids for chronic pain: A review of the place of oxycodone/naloxone. *Clin Drug Investig* 2015;35(1):1-11.
14. Lazzari M, Greco MT, Marcassa C, *et al*. Efficacy and tolerability of oral oxycodone and oxycodone/ naloxone combination in opioid-naïve cancer patients: A propensity analysis. *Drug Des Devel Ther* 2015; 9: 5863-72.
15. Camilleri M. Opioid-induced constipation: Challenges and therapeutic opportunities. *Am J Gastroenterol* 2011;106(5):835-42.
16. Rauck R, Slatkin NE, Stambler N, et al. Randomized, Double-Blind Trial of Oral Methylnaltrexone for the Treatment of Opioid-Induced Constipation in Patients with Chronic Noncancer Pain. Pain Practice. *Pain Pract* 2017;17(6):820-28.
17. Alame AM, Bahna H. Evaluation of constipation. *Clin Colon Rectal Surg* 2012; 25(1):5–11.
18. Drewes AM, Munkholm P, Simrén M, *et al*. Definition, diagnosis and treatment strategies for opioid-induced bowel dysfunction—Recommendations of the Nordic Working Group. *Scand J Pain* 2016;11:111-22.
19. Muller-Lissner S, Bassotti G, Coffin B, *et al*. Opioid-induced constipation and bowel dysfunction: a clinical guideline. *Pain Med* 2016; 18(10): 1837–63.
20. Farmer AD, Holt CB, Downes TJ, *et al*. Pathophysiology, diagnosis, and management of opioid-induced constipation. *Lancet Gastroenterol Hepatol* 2018;3(3):203-12.
21. Slatkin N, Thomas J, Lipman AG, *et al.* Methylnaltrexone for treatment of opioid-induced constipation in advanced illness patients. *J Support Oncol* 2009; 7(1): 39–46.
22. Zhou SL, Zhang XL, Wang JH. Comparison of electroacupuncture and medical treatment for functional constipation: a systematic review and meta-analysis. *Acupunct Med* 2017;35(5):324-31._
23. Zhang T, Chon TY, Liu B, *et al*. Efficacy of acupuncture for chronic constipation: a systematic review. *Am J Chin Med* 2013;41(4):717-42.
24. Liu Z, Yan S, Wu J, *et al*. Acupuncture for Chronic Severe Functional Constipation: A Randomized Trial. *Ann Intern Med* 2016;165(11):761-69.

25. Liu B, Wu J, Yan S, et al. Electroacupuncture vs Prucalopride for Severe Chronic Constipation: A Multicenter, Randomized, Controlled, Noninferiority Trial. Am J Gastroenterol. 2021;116(5):1024-1035.
26. Takahashi T. Mechanism of acupuncture on neuromodulation in the gut–a review. *Neuromodulation*. 2011;14:8-12; discussion 12
27. Oken MM, Creech RH, Tormey DC, *et al*. Toxicity and response criteria of the Eastern Cooperative Oncology Group. *Am J Clin Oncol* 1982;5(5): 649-55.
28. Lewis SJ, Heaton KW. Stool form scale as a useful guide to intestinal transit time. *Scand J Gastroenterol* 1997;32(9):920-4.
29. Katakami N, Oda K, Tauchi K, et al. Phase IIb, Randomized, Double-Blind, Placebo-Controlled Study of Naldemedine for the Treatment of Opioid-Induced Constipation in Patients With Cancer. *J Clin Oncol*. 2017;35(17):1921-8.
30. Song Yulei, Lin Zheng, Lin Lin, *et al*. Validation of the Chinese version of PAC-SYM questionnaire. *Journal of Nursing Science* 2012;27(7):73-6.
31. Marquis P, De La Loge C, Dubois D, *et al*. Development and validation of the Patient Assessment of Constipation Quality of Life questionnaire. *Scand J Gastroenterol* 2005;40(5): 540-51.
32. Xun Jin, Yi-Jiang Ding, Shu-Qing Ding, *et al*. Evaluation of the Chinese version of the Patient Assessment of Constipation Quality of Life scale. *World Chinese Journal of Digestology* 2011, 19(2): 209-13.
33. Preston CC, Colman AM. Optimal number of response categories in rating scales: reliability, validity, discriminating power, and respondent preferences. *Acta Psychol*. 2000;104(1):1–15.
34. Standardization Administration of the People's Republic of China. GB/ T12346-2006, Nomenclature and Location of Acupuncture Points [S].2006.

12 Major update of the published protocol

Important Changes Made to Study Design After Trial Commencement

No.	Item	Original version	Final version	Reason(s) for making change(s)
1	Exclusion criteria	(1)Patients diagnosed with clinically significant abnormal defecation due to functional disorders or structural abnormalities of the gastrointestinal tract and other tissues related to gastrointestinal tract (not including OIC)	Patients diagnosed with clinically significant abnormal defecation due to structural abnormalities of the gastrointestinal tract and other tissues related to gastrointestinal tract (not including OIC)	To reconsider the appropriate exclusion for more enrollment
2	Exclusion criteria	(13)Patients that received an opioid receptor antagonist or agonist within one month of the screening, or those who are scheduled to receive such therapy during the study	Patients that received an opioid receptor antagonist one month before the screening, or those who are scheduled to receive such therapy during the study	To reconsider the appropriate exclusion for more enrollment
3	Primary outcome	The primary outcome will be the proportion of responders, ...	The primary outcome will be the proportion of **overall** responders, ...	To make this point more clearly

Effects of Electroacupuncture on Opioid-induced Constipation in Patients with Cancer: A Randomized Controlled Trial

Statistical Analysis Plan

(Final 1.1)

Dongzhimen Hospital, Beijing University of Chinese Medicine

10 December, 2021

CONTENTS

1. Introduction 2
2. Study objective 3
3. Design 3
4. Statistical Considerations 3
    4.1 Study Hypothesis 3
    4.2 Statistical Hypothesis 3
    4.3 Study Populations 3
    4.4 Statistical Analyses 3
        4.4.1 The General Principle 3
        4.4.2 Demographics and Baseline Characteristics 4
        4.4.3 Analyses for Primary Outcome 4
        4.4.4 Analyses for Secondary Outcome 4
        4.4.5 Safety Analyses 5
5. The Summary of Changes of Final SAP 6

1. Introduction

About 70-80% of patients experience moderate to severe pain. As the cornerstone of treatment for moderate to severe cancer pain, opiate analgesics, such as morphine and oxycodone, are recommended by WHO Cancer Pain Relief Guidelines. The use of systemic opioids is recommended by some studies for cancer patients experiencing moderate to severe pain, regardless of the underlying causes. Opioids stimulate receptors both in the central nervous system (CNS) and the peripheral nervous system, reducing pain and improving quality of life for patients. The drug can, however, be associated with serious adverse events (AEs) with a rate ranging from 1.8% to 13.6%, the most common of which is opioid-induced constipation (OIC). OIC represents a change in baseline bowel habits or defecation patterns that occurs following the administration or modification of opioid therapy. Approximately 41% of non-cancer patients and 94% of cancer patients who use opioids for pain have this condition. Symptoms of OIC are usually persistent and difficult to tolerate, which adversely affects patients' quality of life and results in reductions in dose or discontinuation of opioid analgesics. OIC is the result of multiple factors contributing to it: Opioids may activate μ-receptors throughout the gastrointestinal tract and cause changes to gut motility, decreases in gut secretion, and an increase in sphincter tone, which can lead to constipation. Various pharmacological and nonpharmacological interventions are used to manage OIC, such as laxatives and increased fluid intake. However, these interventions are limited in effectiveness, and they do not address the pathophysiological mechanisms of OIC. However, longer-term efficacy and safety of PAMORAs are unclear, and they haven't been approved in China yet. Clinical trials are still underway to test these drugs. Additionally, PAMORAs are often associated with AEs such as abdominal pain and flatulence. As a result, it is still necessary to explore new treatment approaches for OIC.

Acupuncture has been used to treat gastrointestinal disease, including constipation, for thousands of years. According to two systematic reviews, acupuncture can improve spontaneous bowel movements (SBMs) in functional constipation. Additionally, the results of our study indicated that electroacupuncture (EA) could increase complete spontaneous bowel movements (CSBMs) and SBMs, with a long-term effect that continues for 24 weeks after treatment ceased among patients with chronic, severe

functional constipation. Through stimulation of the somatic and peripheral nervous systems, acupuncture can facilitate the gut motility and improve gastrointestinal function. The effectiveness of acupuncture for OIC is currently lacking evidence.

2. Study objective

The objective of this study is to assess the efficacy of EA for OIC in adult patients with cancer pain.

3. Design

This is a multicenter, sham-controlled, assessor-blinded, randomized trial.

4. Statistical Considerations

4.1 Study Hypothesis

The primary study hypothesis is that EA is more effective than SA in patients with cancer pain.

4.2 Statistical Hypothesis

The null hypothesis is that the proportion of overall responders will be the same for EA and SA, and the alternative hypothesis is that the change would differ.

4.3 Study Populations

All patients with randomization will be included in the analysis set regardless of whether they receive any treatment. According to the intention-to-treat principle, all analysis will be based on the randomization set.

4.4 Statistical Analyses

4.4.1 The General Principle

Summary tables (descriptive statistics and/or frequency tables) will be provided for all variables at different endpoints. For continuous variables, means and standard deviations will be presented, unless the variable has a skewed distribution, in which case medians, 25th and 75th percentiles will be presented. For categorical variables, the number and percentage of participants within each category will be presented. For each variable (continuous or categorical), the number of missing values will be reported.

Statistical Comparisons Between Groups

Continuous variables will be compared using a two-sample *t*-test or Wilcoxon rank-sum test if data show serious deviations from a normal distribution. Categorical data or ordinal data will be compared using a Wilcoxon rank-sum test, chi-square test or Fisher's exact test, as appropriate. All tests will be two-sided.

For the analysis of the primary and secondary outcomes, estimated treatment differences and associated 95% two-sided confidence intervals will be presented.

Analysis Software

For all statistical analyses, SAS 9.4 software will be used. All hypothesis testing will be carried out at the 5% (2-sided) significance level.

4.4.2 Demographics and Baseline Characteristics

All data recorded at baseline will be summarized by group. Comparisons between groups will be performed using the methodology described in section 4.4.1. Summaries will be presented for the ITT Set in both groups.

4.4.3 Analyses for Primary Outcome

The primary outcome will use a generalized linear model with a binomial distribution and identity link. The subgroup analysis will be conducted by adding an interaction between the baseline daily opioid dose and treatment into the generalized linear model.

Missing data on the primary outcome will be imputed using the multiple imputation method under the missing at random assumption.

4.4.4 Analyses for Secondary Outcomes

Efficacy analyses for all secondary outcomes will be performed in the ITT population, without imputation of missing data.

Continuous data will be described with the average, standard deviation, median, minimum value, and maximum value, whereas categorical data will be represented by percentages as appropriate.

4.4.5 Safety Analyses

All adverse events and serious adverse events will be listed. Adverse events include the acupuncture-related adverse events and other adverse events.

5. The Summary of Changes of Final SAP

As compared to the original protocol published in the *Front. Med.* (Zhishun Liu, Yang Wang, Huanfang Xu, et al. Effects of Electroacupuncture on Opioid-Induced Constipation in Patients with Cancer: Study Protocol for a Multicenter Randomized Controlled Trial. Front Med. 2022), the present finalized SAP had made a few amendments. The major updates were provided in Table 1.

Table 1. MAJOR UPDATES OF THE ORIGINAL SAP

No.	Item	Original Version	Final Version
1	Primary outcome	The primary outcome will be evaluated using **the x^2 test.**	The primary outcome will use **a generalized linear model** with a binomial distribution and identity link.
2	Safety outcome	AE incidences for each treatment group will be compared using **Fisher's exact test**.	AE data will be provided for **descriptive purposes only.**

Ethical Approvals of all participating hospitals

This trial is to be conducted in 7 hospitals. The ethical review was firstly submitted to IRB of the principle organization, Guang'an men Hospital, and then to IRB of other participating hospitals. This trial has gained approval from all of the IRBs.

As both Wangjing Hospital and Guang'an men Hospital are affiliated to China Academy of Chinese Medical Sciences, Wangjing Hospital shared the same ethical approval as the Guang'an men Hospital.

Ethical approvals are attached in the following sequence.

1. Guang'an men Hospital Affiliated to China Academy of Chinese Medical Sciences
2. The First Affiliated Hospital of Guizhou University of Traditional Chinese Medicine
3. Zhejiang Hospital
4. Jiangsu Province Hospital of Traditional Chinese Medicine
5. Hengyang Hospital affiliated with Hunan University of Chinese Medicine
6. Yantai Hospital of Traditional Chinese Medicine

Institutional Review Board Documentation of Guang'anmen Hospital of China Academy of Chinese Medical Sciences (EC_AF_054)

Ethical Approvals of Guang' anmen Hospital of China Academy of Chinese Medical Sciences

Trial name	Effect of acupuncture for opioid-induced constipation in patients with cancer: a randomized controlled trial		
Approval No.	2018-164-KY-01	Project Sponsor	Investigator
Participating Centers	Guang'an men Hospital Affiliated to China Academy of Chinese Medical Sciences,The First Affiliated Hospital of Guizhou University of Traditional Chinese Medicine, Zhejiang Hospital, Jiangsu Province Hospital of Traditional Chinese Medicine, Hengyang Hospital affiliated with Hunan University of Chinese Medicine, Wangjing Hospital affiliated to China Academy of Chinese Medical Sciences, Yantai Hospital of Traditional Chinese Medicine		
Site PI	Zhishun Liu	Research department	Acupuncture and Moxibustion Deparment
Review Attribute	Second review	Review methods	Quick Review
Review Date	December 20, 2018	Review Place	Guang'an men Hospital Affiliated to China Academy of Chinese Medical Sciences
Committee Member	Haibo Yin, Wei Cao		
Approval Files	Study Protocol（VERSION1.0_ 2018093001/September 30, 2018）, Case Report Form(VERSION1.0_ 2018093001/September 30, 2018) Informed Consent（VERSION1.0_ 2018093001/September 30, 2018）, Participant recruitment advertisement (VERSION1.0_ 2018093001/September 30, 2018), Diary card in screening period (VERSION1.0_ 2018093001/September 30, 2018), Diary card in treatment period (VERSION1.0_ 2018093001/September 30, 2018), Diary card in follow-up period (VERSION1.0_ 2018093001/September 30, 2018), Investigator's Brochure(VERSION1.0_ 2018093001/September 30, 2018), Emergency plan(VERSION1.0_ 2018093001/September 30, 2018).		
Review Comments	According to "ethical review methods for biomedical study involving human subjects" issued by the Ministry of Health, "Good Clinical Practice", "Provisions for Clinical Trials of Medical Device" and "Guidelines for Ethical Review Work of Drug Clinical Trials" issued by State Food and Drug Administration (SFDA) of the People's Republic of China, "management specifications for ethical review of TCM clinical studies" issued by State Administration of Traditional Chinese Medicine, "Declaration of Helsinki", and "International ethical guidelines for biomedical research involving human subjects" issued by Council for International Organizations of Medical Sciences, this clinical research was reviewed by the institutional review board (IRB) of Guang'anmen Hospital of China Academy of Chinese Medical Sciences. And the		

	study protocol, informed consent, and the recruitment files of this research were approved.<br>Please conduct this clinical study following the GCP principles and the study protocol approved by the IRB. The health and rights of the subjects should be protected throughout the whole study.<br>This approval will be invalid if the trial could not be initiated within three years, and the application should be resubmitted.<br>An application should be submitted if a change of the principle investigator (PI), or any modification of the study protocol, informed consent, or the recruitment files are made.<br>A report of the severe adverse events (SAE) should be submitted within 15 working days if any SAE or any other un-anticipated AE, which will affect the risk-reward ratio of this study, occurs. If a fatal adverse event occurs, please submit a serious adverse event report as soon as it becomes known.<br>Researchers should submit report of the study progress one month before the deadline according to ethical review frequency. A summary report of the study progress of each site should be submitted by the site PI to the IRB of the leading site. In any condition which will greatly affect the progress of the study or increase the potential risk of the subjects, a written report should be submitted by the site PI to the IRB.<br>A protocol deviation report should be submitted by the site PI/monitor/researcher if any of the following occurs: conditions that violate the study protocol: subjects who did not meet the inclusion criteria, or should be excluded according to the exclusion criteria, were wrongly included in the study; incorrect treatment or dose was given; prohibited combined medicine was used; conditions that violate GCP principle: subjects' rights and health are badly affected; the science of study was badly affected.<br>A final report should be submitted when the study is finished completely or terminated prematurely.		
Validity Period	From December 25,2018 to December 24, 2019		
Tracking review frequency	12 months	Tracking review date	December 24, 2019
Contact	Jie Qiao, +86 010-88001552, E-mail: gamhec@126.com		
Director Signature	Haibo Yin		
IRB of Guang'anmen Hospital of China Academy of Chinese Medical Sciences (Seal)			
Date: December 24, 2018			

Version No.03.05 / Version Date: 20170824

伦理审查批件

项目名称	电针缓解癌痛阿片类药物性便秘--随机对照试验		
批件号	2018-164-KY-01	项目来源	研究者
研究单位	中国中医科学院广安门医院		
申办者	无		
主要研究者	刘志顺	研究科室	针灸科
审查类别	复审	审查方式	快速审查
审查日期	2018-12-20	审查地点	中国中医科学院广安门医院
审查委员	殷海波，曹炜		
批准文件及版本	1. 研究方案（版本号：2018093001；版本日期：2018-09-30）<br>2. 病例报告表（版本号：VERSION 1.0_20180930；版本日期：2018-09-30）<br>3. 知情同意书（版本号：2018113001；版本日期：2018-11-30）<br>4. 招募广告（版本号：20180930；版本日期：2018-09-30）<br>5. 日记卡-筛选期（版本号：VERSION 1.0_20180930；版本日期：2018-09-30）<br>6. 日记卡-治疗期（版本号：VERSION 1.0_20180930；版本日期：2018-09-30）<br>7. 日记卡-随访期（版本号：VERSION 1.0_20180930；版本日期：2018-09-30）<br>8. 研究者手册（版本号：2018093001；版本日期：2018-9-30）<br>9. 应急预案（版本号：2018113001；版本日期：2018-11-30）		
审查意见	根据国家卫生计生委《涉及人的生物医学研究伦理审查办法》、国家药品食品监督管理局《药物临床试验质量管理规范》、《医疗器械临床试验规定》、《药物临床试验伦理审查工作指导原则》国家中医药管理局《中医药临床研究伦理审查管理规范》以及《赫尔辛基宣言》和国际医学科学组织委员会颁布的《人体生物医学研究国际道德指南》的伦理原则，经本伦理委员会审查，同意按所批准的临床研究方案、知情同意书、招募材料开展本项研究。<br>请遵循 GCP 原则、遵循伦理委员会批准的方案开展临床研究，保护受试者的健康与权利。<br>若在三年内未启动研究，本批件作废，需重新提交伦理审查申请。<br>研究过程中若变更主要研究者，对临床研究方案、知情同意书、招募材料等的任何修改，请申请人提交修正案审查申请。<br>如发生严重不良事件以及影响研究风险受益比的非预期不良事件，请申请人在获知后15个工作日内提交严重不良事件报告，如果是致死的不良反应，请在获知后立即提交严重不良事件报告。<br>请按照伦理委员会规定的年度/定期跟踪审查频率，申请人在截止日期前1个月提交研究进展报告；申办者应当向组长单位伦理委员会提交各中心研究进展的汇总报告；当出现任何可能显著影响试验进行或增加受试者危险的情况时，请申请人及时向伦理委员会提交书面报告。<br>研究纳入了不符合纳入标准或符合排除标准的受试者，符合中止试验规		

	定而未让受试者退出研究，给予错误治疗或剂量，给予方案禁止的合并用药等没有遵从方案开展研究的情况；或可能对受试者的权益/健康以及研究的科学性造成不良影响等违背 GCP 原则的情况，请申办者/监查员/研究者提交违背方案报告。<br>提前终止或完成临床研究，请及时提交研究完成报告。		
批件有效期	2018 年 12 月 25 日～2019 年 12 月 24 日		
跟踪审查频率	12 个月	跟踪审查截止日期	2019 年 12 月 24 日
联系人与联系方式	联系人：乔洁　　联系电话：010-88001552　　Email：gamhec@126.com		
主任委员/副主任委员签字			
中国中医科学院广安门医院伦理委员会（盖章）			
日期：2018 年 12 月 [illegible] 日			

Ethical Approvals of the First Affiliated Hospital of Guizhou University of Traditional Chinese Medicine

Approval No.	H2019-001		
Trial name	Effect of acupuncture for opioid-induced constipation in patients with cancer: a randomized controlled trial		
Project source	Guang'an men Hospital Affiliated to China Academy of Chinese Medical Sciences		
Applicators	Cunxia LU		
Review Date	March 5, 2019	Review Place	GCP meeting room in the First Affiliated Hospital of Guizhou University of Traditional Chinese Medicine
Approval Files	Technical Service Contract, Ethical Approvals of Guang' anmen Hospital of China Academy of Chinese Medical Sciences		
Review Comments	According to "ethical review methods for biomedical study involving human subjects(2016)" issued by the Ministry of Health, "Good Clinical Practice", "International ethical guidelines for biomedical research involving human subjects" issued by Council for International Organizations of Medical Sciences, and "Declaration of Helsinki", this research was approved after review by the ethics committee.<br>Please conduct the study in accordance with the protocol approved by the ethics committee, and protect the health and rights of the participants.		
Ethics Comm ittee	IRB of he First Affiliated Hospital of Guizhou University of Traditional Chinese Medicine (Seal)		
Date	March 5, 2019		

贵阳中医学院第一附属医院伦理委员会

伦理审查批件（科研）

批件号	H2019-001		
项目名称	电针缓解癌痛阿片类药物性便秘—随机对照试验		
项目来源	中国中医科学院广安门医院		
申请人	卢春霞		
审查日期	2019.03.05	审查地点	贵阳中医学院第一附属医院 GCP 会议室
审查文件	技术服务合同、北京总中心的伦理批件		

审查意见：

根据卫计委《涉及人的生物医学研究伦理审查办法（2016）》、WMA《赫尔辛基宣言》和CIOMS《人体生物医学研究国际道德指南》的伦理原则，经本伦理委员会审查，同意开展本项科研研究。

请遵循伦理委员会批准的方案开展研究，保护受试者的健康与权力。

伦理委员会（盖章）	贵阳中医学院第一附属医院伦理委员会
日期	2019.03.05

49

Approval letter of Medical Ethics Committee of Zhejiang Hospital

Approval No.: 2019-PI-(6k)　　　　Date of issue: 2019. 2. 28

Study title	Effect of acupuncture for opioid-induced constipation in patients with cancer: a randomized controlled trial		
Sponsor	Zhejiang Hospital		
Principal investigator	Xiaoqing Jin, Jianfang Zhu	Specialty	Acupuncture and moxibustion department
Category of review	Initial review, second review	Type of review	Meeting review, quick review
Date of review	2019.2.22, 2019.2.28	Location of review	Meeting room131 in No. 8 building
Reviewed items	1. Application form for Initial Review of the study<br>2. Study Protocol（VERSION1.0_ 2018093001/September 30, 2018）<br>3. Supplementary Study Protocol of Zhejiang Hospital（VERSION1.0, January 3, 2019）<br>4. Informed Consent（VERSION2.1, February 27, 2019）<br>5. Participant recruitment advertisement（VERSION1.0, January 3, 2019）<br>6. Emergency plan<br>7. Case Report Form(VERSION1.0_ 2018093001/September 30, 2018)<br>8. Diary card in screening period (VERSION1.0_ 2018093001/September 30, 2018)<br>9. Diary card in treatment period (VERSION1.0_ 2018093001/September 30, 2018)<br>10. Diary card in follow-up period (VERSION1.0_ 2018093001/September 30, 2018)<br>11.The resume of principal investigator and certification of GCP<br>12. Ethical Approvals of Guang' anmen Hospital of China Academy of Chinese Medical Sciences		
Evaluation	The ethics committee conducted a meeting review and rapid review of the above items, and believed that the investigator's qualification, clinical study plan, informed consent, recruitment advertisement and benefit and risk assessment were basically in compliant with the ethical requirements , and agreed to carry out the clinical study.		
Decision	The Committee's decision on the study protocol: Approval		
Continual review	Will be the study accept a follow-up review during the study by the Ethics committee? Yes<br>The frequency of review will be once every 12 months from the date of approval of the study, please submit the research progress report one month before February 27, 2020.<br>The ethics committee reserves the right to change the frequency of follow-up reviews based on actual progress.		

Notes:
1. Please follow the relevant laws and regulations of China, “ Standard for quality management of drug clinical trials(2003)” and “ Standard for quality management of clinical trials on medical devices(2016)” issued by China Food and Drug Administration (CFDA), Declaration of Helsinki”,, and “International ethical guidelines for biomedical research involving human subjects” issued by Council for International Organizations of Medical Sciences, “ethical review methods for biomedical study involving human subjects(2016)” issued by the Ministry of Health.
2. Please follow the clinical study protocol, informed consent and recruitment materials approved by the ETHICS Committee to conduct this study, and protect the health and rights of the participants. Any changes to the study protocol, informed consent and recruitment materials must be reviewed and approved by the ethics committee.
3. Serious adverse events or unexpected adverse events occurring in Zhejiang hospital should be submitted to our ethics committee within 24 hours. Serious adverse events or unexpected adverse events occurring at other centers in China shall be periodically collected and submitted to the ethics committee. All unexpected adverse events occurring in the foreign branch centers shall also be periodically collected and submitted to the ethics committee, the ethics committee reserves the right to make a new decision on its assessment.
4. Starting today, whether the trial has started or not, a progress report is required one month before the follow-up review is due. If the study is in progress, please submit the study progress report 2 months before the approval expires. The study can be continued only after the approval is reviewed and approved by the ethics committee.
5. The investigator and sponsor shall submit a summary of the center's research progress report to the ethics committee of the leader site, and the applicant shall timely submit a written report to the ETHICS Committee in case of any situation that may affect the conduct of the study or increase the risk of subjects.
6. A protocol deviation report should be submitted by the site investigator and sponsor if any of the following occurs: conditions that violate the study protocol: subjects who did not meet the inclusion criteria, or should be excluded according to the exclusion criteria, were wrongly included in the study; incorrect treatment or dose was given; prohibited combined medicine was used; conditions that violate GCP principle: subjects’ rights and health are badly affected; the science of study was badly affected.
7. If the applicant suspends or terminates the clinical study in advance, please submit the suspension or termination report in time.
8. A final report should be submitted when the study is finished completely or terminated prematurely.

Chair signature	He Xiaobo
Approval date	2019.2.28
Stamp of ZJEC	Ethics Committee of Zhejiang Hospital(seal)
Period of validity	This approval is valid from February 28, 2019, to February 27, 2022. If it is not implemented within the time limit, it shall be abolished automatically
Statement	The responsibilities, personnel composition, operating procedures and records of the ethics committee have followed the ethical review principle of “Quality management standards for drug clinical trials(2003)”, “ Standard for quality management of clinical trials on medical devices(2016)” and ICH-GCP promulgated by the National Medical Products Administration of the People's Republic of China, and abide by the relevant laws and regulations of China.

Address: No 12, Linyin road, Hangzhou, Zhejiang, China (310013), Contact: +86 0571-81595231, Contact person: WeiLi/Xiaoping Xie, E-mail: zjyykjkli@163.com

54

浙江医院医学伦理委员会 临床试验审查批件

Approval Letter of Medical Ethics Committee of Zhejiang Hospital

批件号 Approval NO.：2019 临审第（6K）号　　　　**签发日期 Date of issue ： 2019.02.28**

**项目名称**<br>**Study Title**	电针缓解癌痛阿片类药物性便秘——随机对照试验临床研究		
**申办方**<br>**Sponsor**	浙江医院		
**主要研究者**<br>**Principal Investigator**	金肖青、诸剑芳	**承担专业**<br>**Specialty**	针灸科
**审查类别**<br>**Category of Review**	初始审查、复审	**审查方式**<br>**Type of Review**	会议审查、快速审查
**审查日期**<br>**Date of Review**	2019.02.22、2019.02.28	**审查地点**<br>**Location of Review**	8 号楼 313 会议室<br>各自办公室
**审查文件清单**<br>**Reviewed Items**	1. 临床课题研究初始审查申请书<br>2. 研究方案，版本号：2018093001，日期：2018.9.30<br>3. 浙江医院补充临床研究方案，1.0 版，日期：2019.01.03<br>4. 知情同意书，2.1 版，日期：2019.02.27<br>5. 招募广告，2.0 版，日期：2019.01.03<br>6. 应急预案<br>7. 病例报告表，版本号：VERSION 1.0_20180930<br>8. 患者日记卡（筛选期），版本号和日期：VERSION 1.0_20180930<br>9. 患者日记卡（随访期），版本号和日期：VERSION 1.0_20180930<br>10. 患者日记卡（治疗期），版本号和日期：VERSION 1.0_20180930<br>11. 主要研究者履历及 GCP 证书<br>12. 研究者责任声明<br>13. 组长单位伦理批件		
**审评意见**<br>**Evaluation**	本伦理委员会对上述资料进行了会议审查和快速审查，认为研究者资质、临床研究方案、知情同意书、招募广告和受益与风险评估等基本符合伦理规范，同意实施临床研究。		
**审查决定**<br>**Decision**	委员会对该方案的审查决定为：　■同意（Approval）		
**年度/定期跟踪审查**<br>**Continual Review**	该研究进行过程中将接受伦理委员会的跟踪审查？■是(Yes)　　□否(No)<br>审查频率为该研究批准之日起每 12 个月一次，首次，请于 2020 年 02 月 27 日前 1 个月递交研究进展报告。<br>伦理委员会有根据实际进展情况改变跟踪审查频率的权利。		

地址：杭州市灵隐路 12 号　邮编：310013　电话：0571-81595231　联系人：李卫/谢小萍　邮箱：zjyykjkli@163.com

备注：

1. 请遵循我国相关法律、法规和规章 CFDA《药物临床试验质量管理规范（2003）》、《医疗器械临床试验质量管理规范（2016）》、WMA《赫尔辛基宣言》和 CIOMS《人体生物医学研究国际道德指南》和国家卫生健康委员会《涉及人的生物医学研究伦理审查办法（2016）》的伦理原则。
2. 请遵循经本伦理委员会批准的临床研究方案、知情同意书、招募材料开展本研究，保护受试者的健康与权利。对研究方案、知情同意书和招募材料等的任何修改，均须得到伦理委员会审查同意后方可实施。
3. 在浙江医院发生的严重不良事件或非预期不良事件应 24 小时内递交本医学伦理委员会，国内其他中心发生的严重不良事件或非预期不良事件需定期汇总后递交本伦理委员会，对于国外发生的非预期不良事件定期汇总后递交伦理委员会，伦理委员会有权对其评估做出新的决定。
4. 自今日起，无论试验开始与否，请在跟踪审查到期前 1 个月提交研究进展报告；若研究正在进行中，请在批件到期前 2 个月，递交研究进展报告，须得到伦理委员会审查同意延长批件有效期后方可继续进行。
5. 研究者和申办方应当向组长单位伦理委员会提交中心研究进展报告汇总；当出现任何可能显著影响试验进行或增加受试者危险的情况时，请申请人及时向伦理委员会提交书面报告。
6. 研究纳入了不符合纳入标准或符合排除标准的受试者，符合中止试验规定而未让受试者退出研究，给予错误治疗或剂量，给予方案禁止的合并用药等没有遵从方案开展研究的情况；或可能对受试者的权益或健康以及研究的科学性造成不良影响等违背 GCP 原则的情况，请研究者和申办者提交违背方案报告。
7. 申请人暂停或提前终止临床研究，请及时提交暂停或终止研究报告。
8. 完成临床研究，请申请人提交结题报告。

主任签字<br>Chair Signature	[illegible]
批准日期<br>Approval Date	2019.2.28
伦理委员会<br>Stamp of ZJEC	浙江医院医学伦理委员会（盖章）<br>浙江医院医学伦理委员会
批件有效期<br>Period of Validity	此批件的有效期为（2019.02.28-2022.02.27），逾期未实施的，自行废止。
声明<br>Statement	本伦理委员会的职责、人员组成、操作程序及记录遵循中华人民共和国药品监督管理局颁布的药物临床试验质量管理规范（2003）、医疗器械临床试验质量管理规范（2016）和 ICH-GCP 的伦理审查原则，并遵守中国的相关法律及法规。

59 ## Approvals of Ethical Review

Approval No.	2019NL-031-03		
Trial name	Effect of acupuncture for opioid-induced constipation in patients with cancer: a randomized controlled trial		
Project source	Guang'an men Hospital Affiliated to China Academy of Chinese Medical Sciences		
Participating Center	Jiangsu Province Hospital of Traditional Chinese Medicine		
Site PI	Jianhua Sun		
Review Attribute	Second review	Review methods	Quick Review
Review Date	June 18, 2019	Review Place	
Committee Member	Yuhong Xu		
Approval Files	Revised study Protocol（ VERSION_ 2019040901/April 9, 2019 ）, Revised participant recruitment advertisement (VERSION_ 2019040901/April 9, 2019), Revised informed Consent(VERSION_ 2019040901/April 9, 2019)		
Review Comments	According to "ethical review methods for biomedical study involving human subjects(2016)" issued by the Ministry of Health, "Good Clinical Practice(2003)", "Provisions for Clinical Trials of Medical Device(2016)", "Declaration of Helsinki" and "International ethical guidelines for biomedical research involving human subjects" issued by Council for International Organizations of Medical Sciences, this clinical research was reviewed by the institutional review board (IRB) of Guang'anmen Hospital of China Academy of Chinese Medical Sciences. And the study protocol, informed consent, and the recruitment files of this research were approved.<br>Please conduct this clinical study following the GCP principles and the study protocol approved by the IRB. The health and rights of the subjects should be protected throughout the whole study.<br>Prior to the implementation of a research project approved by the ethics committee, the principal of the research project shall register the main contents and ethical review decisions of the research project in the medical research Registration and archival Information system. Research projects involving Chinese human genetic resources that need to be submitted for approval should be approved by the Chinese Office of Human Genetic Resources Management before starting research.<br>An application should be submitted if a change of the principle investigator (PI), or any modification of the study protocol, informed consent, or the recruitment files are made.<br>If a serious adverse event occurs, the applicant should submit a serious adverse event report in time<br>Researchers should submit report of the study progress one month before the deadline according to ethical review frequency. A summary report of the study progress of each site should be submitted by the site PI to the IRB of the leading site. In any condition which will greatly affect the progress of the study or increase the potential risk of the subjects, a written report should be submitted by the site PI to the IRB. Beyond the period of validity, if research progress report of the study project was not submitted		

	and the study was not obtaining ethical approval to continue the study project, researchers must immediately stop all research activities, including intervention and data collection. If discontinuing the study intervention could cause harm to the subject, the investigator should ask the ethics committee to approve the continuing study of the subject.<br>A protocol deviation report should be submitted by the site PI/monitor/researcher if any of the following occurs: conditions that violate the study protocol: subjects who did not meet the inclusion criteria, or should be excluded according to the exclusion criteria, were wrongly included in the study; incorrect treatment or dose was given; prohibited combined medicine was used; conditions that violate GCP principle: subjects' rights and health are badly affected; the science of study was badly affected.<br>If the applicant has suspended or terminated the clinical study in advance, please submit the suspension/termination report timely.<br>To complete the clinical study, applicants shall submit a study completion report and a summary report outlining the study findings and conclusions.
Annual/Regular tracking review frequency	Please submit the research progress report one month before June 18, 2020
Validity Period	12 months
Contact	Jing Wu, +86 025-86560515
Director Signature	Ming hua Wu
IRB of 4. Jiangsu Province Hospital of Traditional Chinese Medicine of Nanjing University of Traditional Chinese Medicine (Seal)	
Date: June 18, 2019	

伦理审查批件

批件号	2019NL-031-03		
项目名称	电针缓解癌痛阿片类药物性便秘--随机对照试验		
项目来源	中国中医科学院广安门医院自拟课题		
研究单位	江苏省中医院		
主要研究者	孙建华		
审查类别	复审申请	审查方式	快速审查
审查日期	2019 年 06 月 18 日	审查地点	
审查委员	徐玉红		
审查批准文件	修正的临床研究方案 版本号：2019040901 版本日期：2019-04-09<br>修正的招募材料 版本号：2019040901 版本日期：2019-04-09<br>修正的知情同意书 版本号：2019061201 版本日期：2019-06-12		

审查意见

根据国家卫生计生委《涉及人的生物医学研究伦理审查办法》（2016）、CFDA《药物临床试验质量管理规范》（2003）、《医疗器械临床试验质量管理规范》（2016）、WMA《赫尔辛基宣言》和 CIOMS《涉及人的生物医学研究国际伦理审查指南》的伦理原则，经本伦理委员会审查，同意按所批准的临床研究方案、知情同意书、招募材料开展本项研究。

请遵循 GCP 原则、遵循伦理委员会批准的方案开展临床研究，保护受试者的健康与权利。

经伦理委员会批准的研究项目在实施前，研究项目负责人应当将该研究项目的主要内容、伦理审查决定在医学研究登记备案信息系统进行登记。凡涉及中国人类遗传资源、需要报批的研究项目，应在获得中国人类遗传资源管理办公室批准后才能开始研究。

研究过程中若变更主要研究者，对临床研究方案、知情同意书、招募材料等的任何修改，请申请人提交修正案审查申请。

发生严重不良事件，请申请人及时提交严重不良事件报告。

请按照伦理委员会规定的年度/定期审查频率，申请人在截止日期前 1 个月提交研究进展报告；申办者应当向组长单位伦理委员会提交各中心研究进展的汇总报告；当出现任何可能显著影响试验进行、或增加受试者危险的情况时，请申请人及时向伦理委员会提交书面报告。超出批件有效期，没有提交研究进展报告并获得伦理审查批准继续研究的项目，研究者必须立即停止所有研究活动，包括干预措施和数据收集。假若停止研究干预可能会对受试者造成伤害，研究者应当要求伦理委员会批准在研的受试者继续参加研究。

研究纳入了不符合纳入标准或符合排除标准的受试者，符合中止试验规定而未让受试者退出研究，给予错误治疗或剂量，给予方案禁止的合并用药等没有遵从方案开展研究的情况；或可能对受试者的权益/健康、以及研究的科学性造成不良影响等违背 GCP 原则的情况，请申办者/监查员/研究者提交违背方案报告。

申请人暂停或提前终止临床研究，请及时提交暂停/终止研究报告。

完成临床研究，请申请人提交研究完成报告，以及概述研究发现和结论的总结报告。

年度/定期跟踪审查频率	请于 2020 年 06 月 18 日前 1 个月提交研究进展报告
有效期	12 个月
联系人与联系电话	吴静 025-86560515
主席签字	[illegible]
伦理委员会	南京中医药大学附属医院（江苏省中医院）伦理委员会（盖章）
日期	2019 年 06 月 18 日

Institutional Review Board Document of Hengyang Hospital affiliated with Hunan University of Chinese Medicine (EC-AF-2019006)

Ethical Approvals of Hengyang Hospital affiliated with Hunan University of Chinese Medicine

Trial name	Effect of acupuncture for opioid-induced constipation in patients with cancer: a randomized controlled trial		
Project Sponsor	Guang'an men Hospital Affiliated to China Academy of Chinese Medical Sciences		
Site	Hengyang Hospital affiliated with Hunan University of Chinese Medicine		
Applicant (if any)	/		
Site PI	Zenghui Yue, Jun Xie		
Review Attribute	Initial review	Review Methods	Quick review
Review Date	January 12, 2019	Review Place	Conference room, the 9th floor of hospital clinical building
Committee Member	Chengxi Wang, Shuangcai Long, Yueping Zou, Jiping Xu, Xinlin Zhong, Zhao Kuang, Qiuping Dong		
Approval Files	Study Protocol（VERSION1.0 _ 2018093001）, Informed Consent（VERSION1.0_ 2018113001）		
Review Comments	According to "ethical review methods for biomedical study involving human subjects" issued by the Ministry of Health, "Good Clinical Practice", "Provisions for Clinical Trials of Medical Device" and "Guidelines for Ethical Review Work of Drug Clinical Trials" issued by State Food and Drug Administration (SFDA) of the People's Republic of China, "management specifications for ethical review of TCM clinical studies" issued by State Administration of Traditional Chinese Medicine, "Declaration of Helsinki", and "International ethical guidelines for biomedical research involving human subjects" issued by Council for International Organizations of Medical Sciences, this clinical research was reviewed by the institutional review board (IRB) of the Hengyang Hospital affiliated with Hunan University of Chinese Medicine. And the study protocol, informed consent, and the recruitment files of this research were approved.<br>Please conduct this clinical study following the GCP principles and the study protocol approved by the IRB. The health and rights of the subjects should be protected throughout the whole study.<br>An application should be submitted if a change of the principle investigator (PI), or any modification of the study protocol, informed consent, or the recruitment files are made.<br>A report of the severe adverse events (SAE) should be submitted in time if any SAE or any other un-anticipated AE, which will affect the risk-reward ratio of this study, occurs.<br>Researchers should submit report of the study progress one month before the deadline according to ethical review frequency. A summary report of the study progress of each site should be submitted by the site PI to the IRB of the leading site. In any condition which will greatly affect the progress of the study or increase the potential risk of the subjects, a written report should be submitted by the site PI to the IRB.		

	A protocol deviation report should be submitted by the site PI/monitor/researcher if any of the following occurs: 1) conditions that violate the study protocol: subjects who did not meet the inclusion criteria, or should be excluded according to the exclusion criteria, were wrongly included in the study; subjects do not withdraw from the study when he/she meet the rules of withdrawal; incorrect treatment or dose was given; prohibited combined medicine was used; 2) conditions that violate GCP principle: subjects' rights and health are badly affected; the science of study was badly affected.<br>A final report should be submitted when the study is finished completely or terminated prematurely.
Validity Period	From February, 2019 to February, 2020
Contact	+86 0734-8137737; Jun Xie
Director Signature	Chengxi Wang
Institutional Ethics Committee of Hengyang Hospital affiliated with Hunan University of Chinese Medicine(Seal)	
Date: January 16, 2019	

Version No.1.00/Version Date January 16, 2019 Page 1 of 1

湖南中医药大学附属衡阳医院伦理委员会文件（EC-AF-2019006）

伦理审查批件

项目名称	电针缓解癌痛阿片类药物性便秘—随机对照试验		
项目来源与编号	中国中医科学院基本科研业务费自主选题项目		
牵头单位	湖南中医药大学附属衡阳医院		
申办者（如有）			
主要研究者	岳增辉、谢军		
审查类别	初始审查	审查方式	快速审查
审查日期	2019.01.12	审查地点	医院门诊楼9楼会议室
审查委员	王诚喜、尤自才、邹岳萍、徐慕平、钟跃林、匡耀、董秋萍		
批准文件	研究方案：（版本号：2018093001）<br>知情同意书：（版本号：2018113001）		
审查意见	根据卫生部《涉及人的生物医学研究伦理审查办法（试行）》、国家药品食品监督管理局《药物临床试验质量管理规范》、《医疗器械临床试验规定》、《药物临床试验伦理审查工作指导原则》、国家中医药管理局《中医药临床研究伦理审查管理规范》以及《赫尔辛基宣言》和国际医学科学组织委员会颁布的《人体生物医学研究国际道德指南》的伦理原则，经本伦理委员会审查，统一按所批准的临床研究方案、知情同意书、招募材料开展本项研究。<br>请遵循GCP原则，遵循伦理委员会批准的方案开展临床研究，保护受试者的健康与权力。<br>研究过程中若变更主要研究者，对临床研究方案、知情同意书、招募材料等的任何修改，请申请人提交修正案审查申请。<br>如发生严重不良事件以及影响研究风险受益比的非预期不良事件，请申请人及时提交不良事件报告。<br>请按照伦理委员会规定的年度/定期跟踪审查频率，申请人在截止日期前1个月提交研究进展报告；申报者应当向组长单位伦理委员会提交各中心的研究进展的汇总报告；当出现任何可能显著影响实验进行或增加受试者危险的情况时，请申请人及时向伦理委员会提交书面报告。<br>研究纳入了不符合纳入标准或符合排除标准的受试者，符合中止实验规定而未让受试者退出研究，给予错误治疗或剂量，给以方案禁止的合并用药等没有遵从方案开展研究的情况；或可能对受试者的权益/健康以及研究的科学性造成不良影响等违背GCP原则的情况，请申办者/监察员/研究者提交违背方案报告。<br>提前终止或完成临床研究，请及时提交结题报告。		
有效期	2019年2月-2020年2月		
联系人及电话	谢军：0734-[illegible]		
主任委员签字			
湖南中医药大学附属衡阳医院伦理委员会（盖章）			
2019年1月18日			

共1页/第1页

版本号：1.00/版本　日期：20190118

Ethical Approvals of Yantai Hospital of Traditional Chinese Medicine

<table>
<tr><td>Trial name</td><td colspan="3">Effect of acupuncture for opioid-induced constipation in patients with cancer: a randomized controlled trial</td></tr>
<tr><td>Approval No.</td><td>2018-KY-026</td><td>Project Sponsor</td><td>Investigator</td></tr>
<tr><td>Applicant (if any)</td><td colspan="3">/</td></tr>
<tr><td>Site PI</td><td>Zhiwei Zang</td><td>Research department</td><td>Acupuncture and Moxibustion Deparment</td></tr>
<tr><td>Review Attribute</td><td>Initial review</td><td>Review Methods</td><td>Quick review</td></tr>
<tr><td>Review Date</td><td>December 29, 2018</td><td>Review Place</td><td>Yantai Hospital of Traditional Chinese Medicine</td></tr>
<tr><td>Applicant (if any)</td><td colspan="3">/</td></tr>
<tr><td>Site PI</td><td colspan="3">Zhiwei Zang</td></tr>
<tr><td>Review Attribute</td><td>Second review</td><td>Review Methods</td><td>Quick review</td></tr>
<tr><td>Review Date</td><td>May 15, 2013</td><td>Review Place</td><td>Yantai Hospital of Traditional Chinese Medicine</td></tr>
<tr><td>Committee Member</td><td colspan="3">AIhua Hou, Bo Liang</td></tr>
<tr><td>Approval Files</td><td colspan="3">1. Study Protocol（VERSION1.0_ 2018093001/September 30, 2018）,
2. Case Report Form(VERSION1.0_ 2018093001/September 30, 2018),
3.Informed Consent（VERSION1.0_ 2018093001/September 30, 2018）,
4. Participant recruitment advertisement (VERSION1.0_ 2018093001/September 30, 2018),
5. Diary card in screening period (VERSION1.0_ 2018093001/September 30, 2018),
6.Diary card in treatment period (VERSION1.0_ 2018093001/September 30, 2018),
7. Diary card in follow-up period (VERSION1.0_ 2018093001/September 30, 2018),
8. Investigator's Brochure(VERSION1.0_ 2018093001/September 30, 2018),
9.Emergency plan(VERSION1.0_ 2018093001/September 30, 2018).</td></tr>
<tr><td>Review Comments</td><td colspan="3">According to “ethical review methods for biomedical study involving human subjects” issued by the Ministry of Health, “Good Clinical Practice”, “Provisions for Clinical Trials of Medical Device” and “Guidelines for Ethical Review Work of Drug Clinical Trials” issued by State Food and Drug Administration (SFDA) of the People’s Republic of China, “management specifications for ethical review of TCM clinical studies” issued by State Administration of Traditional Chinese Medicine, “Declaration of Helsinki”, and “International ethical guidelines for biomedical research involving human subjects” issued by Council for International Organizations of Medical Sciences, this clinical research was reviewed by the institutional review board (IRB) of Guang’anmen Hospital of China Academy of Chinese Medical Sciences. And the study protocol, informed consent, and the recruitment files of this research were approved.
Please conduct this clinical study following the GCP principles and the study protocol approved by the IRB. The health and rights of the subjects should be protected throughout the whole study.
This approval will be invalid if the trial could not be initiated within three years, and the</td></tr>
</table>

<table>
<tr><td></td><td colspan="3">application should be resubmitted.
An application should be submitted if a change of the principle investigator (PI), or any modification of the study protocol, informed consent, or the recruitment files are made.
A report of the severe adverse events (SAE) should be submitted within 15 working days if any SAE or any other un-anticipated AE, which will affect the risk-reward ratio of this study, occurs. If a fatal adverse event occurs, please submit a serious adverse event report as soon as it becomes known.
Researchers should submit report of the study progress one month before the deadline according to ethical review frequency. A summary report of the study progress of each site should be submitted by the site PI to the IRB of the leading site. In any condition which will greatly affect the progress of the study or increase the potential risk of the subjects, a written report should be submitted by the site PI to the IRB.
A protocol deviation report should be submitted by the site PI/monitor/researcher if any of the following occurs: conditions that violate the study protocol: subjects who did not meet the inclusion criteria, or should be excluded according to the exclusion criteria, were wrongly included in the study; incorrect treatment or dose was given; prohibited combined medicine was used; conditions that violate GCP principle: subjects' rights and health are badly affected; the science of study was badly affected.
A final report should be submitted when the study is finished completely or terminated prematurely.</td></tr>
<tr><td>Validity Period</td><td colspan="3">From December 30, 2018 to December 29, 2019</td></tr>
<tr><td>Tracking review frequency</td><td>12 months</td><td>Tracking review date</td><td>December 29, 2019</td></tr>
<tr><td>Contact</td><td colspan="3">Jinghua Ma, +86 0535-6597012, E-mail: kjk2127022@163.com</td></tr>
<tr><td>Director Signature</td><td colspan="3">Yunpeng Liu</td></tr>
<tr><td colspan="4">Yantai Hospital of Traditional Chinese Medicine (Seal)</td></tr>
<tr><td colspan="4">Date: December 29, 2018</td></tr>
</table>

烟台市中医医院伦理审查批件

项目名称	电针缓解癌痛阿片类药物性便秘-随机对照试验		
批件号	2018-KY-026	项目来源	研究者
研究单位	烟台市中医医院		
申办者	无		
主要研究者	臧志伟	研究科室	针灸推拿科
审查类别	初始审查	审查方式	快速审查
审查日期	2018-12-29	审查地点	烟台市中医医院
审查委员	侯爱画、梁波		
批准文件及版本	1.研究方案（版本号：2018093001；版本日期：2018-9-30）<br>2.病例报告表（版本号：VEHION 1.0_20180930；版本日期：2018-09-30）<br>3.知情同意书（版本号：2018113001；版本日期：2018-11-30）<br>4.招募广告（版本号：20180930；版本日期：2018-09-30）<br>5.日记卡-筛选期（版本号： VEHION 1.0_20180930；版本日期：2018-09-30）<br>6.日记卡-治疗期（版本号： VEHION 1.0_20180930；版本日期：2018-09-30）<br>7.日记卡-随访期（版本号： VEHION 1.0_20180930；版本日期：2018-09-30）<br>8.研究者手册（版本号：2018093001；版本日期：2018-09-30）<br>9.应急预案（版本号：2018113001；版本日期：2018-11-30）		
审查意见	根据国家卫生计生委《涉及人的生物医学研究伦理审查办法》、国家药品食品监督管理局《药物临床试验质量管理规范》、《医疗器械临床试验规定》、《药物临床试验伦理审查工作指导原则》国家中医药管理局《中医药临床研究伦理审查管理规范》以及《赫尔辛基宣言》和国际医学科学组织委员会颁布的《人体生物学研究国际道德指南》的伦理原则，经本伦理委员会审查，同意按所批准的临床研究方案、知情同意书材料开展本项研究。		

<table>
<tr><td></td><td colspan="3">请遵循 GCP 原则、遵循伦理委员会批准的方案开展临床研究，保护受试者的健康与权利。
若在三年内未启动研究，本批件作废，需重新提交伦理审查申请。
研究过程中若变更主要研究者，对临床研究方案、知情同意书、招募材料等的任何修改，请申请人提交修正审查申请。
如发生严重不良事件以及影响研究风险受益比的非预期不良事件，请申请人在获知后 15 个工作日内提交严重不良事件报告，如果是致死的不良反应，请在获知后立即提交严重不良事件报告。
请按照伦理委员会规定的年度/定期跟踪审查频率，申请人在截止日期前 1 个月提交研究进展报告；申办者应当向组长单位伦理委员会提交各中心研究进展的汇总报告；当出现任何可能显著影响试验进行或增加受试者危险的情况时，请申请人及时向伦理委员会提交书面报告。
研究纳入了不符合纳入标准或符合排除标准的受试者，符合中止试验规定而未让受试者退出研究，给予错误治疗或剂量，给予方案禁止的合并用药等没有遵从方案开展研究的情况；或可能对受试者的权益/健康以及研究的科学性造成不良影响等违背 GCP 原则的情况，请申办者/监查员/研究者提交违背方案报告。
提前终止或完成临床研究，请及时提交研究完成报告。</td></tr>
<tr><td>批件有效期</td><td colspan="3">2018 年 12 月 30 日—2019 年 12 月 29 日</td></tr>
<tr><td>跟踪审查频率</td><td>12 个月</td><td>跟踪审查截止日期</td><td>2019 年 12 月 29 日</td></tr>
<tr><td>联系人与联系方式</td><td colspan="3">联系人:马静华　　联系电话：0535-6597012
Email:kjk2127022@163.com</td></tr>
<tr><td>主任委员/副主任委员签字</td><td colspan="3">刘云鹏</td></tr>
<tr><td colspan="4">烟台市中医医院伦理委员会（盖章）</td></tr>
<tr><td colspan="4">日期：2018 年 12 月 29 日</td></tr>
</table>

烟台市中医医院 伦理委员会

75
