# Supplementary material for: Effects of Electroacupuncture for Opioid-Induced Constipation in Patients With Cancer in China: A Randomized Clinical Trial
Source: JAMA Netw Open. 2023 Feb 22;6(2):e230310. doi: 10.1001/jamanetworkopen.2023.0310 (PMC9947731; doi:10.1001/jamanetworkopen.2023.0310)
Supplement: Supplement 2. — eTable 1. Methods: Trial Inclusion and Exclusion Criteria eFigure. Methods: Location of Acupoints for Both Study Arms eAppendix. Methods: Multiple Imputation and Sensitivity Analyses eTable 2. Results: Patients’ Expectations About the Effectiveness of Acupuncture at Baseline eTable 3. Results: Compliance Data at Week 8 eTable 4. Sensitivity Analyses for the Primary Outcome eTable 5. Results: Subgroup Analyses eTable 6. Results: Weekly SBMs eTable 7. Results: PAC-QOL Total and Subscale Scores eTable 8. Results: Patients’ Global Improvement Assessment, n (%) eTable 9. Results: Blinding Data and Their Association With the Primary Outcome by the Respective Group eTable 10. Results: Change From Baseline in NRS Score for Mean Cancer Pain Intensity and Worst Pain Intensity Within the Preceding Week eTable 11. Results: Proportion of Patients With Change From Baseline in the Dose of Opioid Consumption, n (%) eReferences. [file jamanetwopen-e230310-s002.pdf]

Supplementary Online Content

Wang W, Liu Y, Yang X, et al. Effects of electroacupuncture for opioid-induced constipation in patients with cancer in China: a randomized clinical trial. *JAMA Netw Open.* 2023;6(2):e230310. doi:10.1001/jamanetworkopen.2023.0310

eTable 1. Methods: Trial Inclusion and Exclusion Criteria
eFigure. Methods: Location of Acupoints for Both Study Arms
eAppendix. Methods: Multiple Imputation and Sensitivity Analyses
eTable 2. Results: Patients' Expectations About the Effectiveness of Acupuncture at Baseline
eTable 3. Results: Compliance Data at Week 8
eTable 4. Sensitivity Analyses for the Primary Outcome
eTable 5. Results: Subgroup Analyses
eTable 6. Results: Weekly SBMs
eTable 7. Results: PAC-QOL Total and Subscale Scores
eTable 8. Results: Patients' Global Improvement Assessment, n (%)
eTable 9. Results: Blinding Data and Their Association With the Primary Outcome by the Respective Group
eTable 10. Results: Change From Baseline in NRS Score for Mean Cancer Pain Intensity and Worst Pain Intensity Within the Preceding Week
eTable 11. Results: Proportion of Patients With Change From Baseline in the Dose of Opioid Consumption, n (%)
eReferences.

This supplementary material has been provided by the authors to give readers additional information about their work.

© 2023 Wang W et al. *JAMA Network Open.*

eTable 1. Methods: Trial Inclusion and Exclusion Criteria

Inclusion Criteria	(1) Cancer patients must meet the Rome IV[1] diagnostic criteria for OIC: New or worsening symptoms of constipation following initiation, alteration, or increase in opioid treatment;<br>(2) Patients recruited in this trial must have a history of OIC symptoms for at least 1 week;<br>(3) Patients must be ≥18 years of age and ≤85 years of age;<br>(4) Patient's cancer condition must be stable with a life expectancy that is more than six months;<br>(5) Patients must have an Eastern Cooperative Oncology Group (ECOG) performance status of 0-3;<br>(6) Patients must have been receiving a relatively stable maintained opioid regimen, consisting of a total daily dose of 30 mg to 1000 mg oral morphine equivalents for at least 2 weeks prior to screening for cancer pain. Furthermore, it must be anticipated that the opioid will be maintained for at least 10 weeks;<br>(7) The SBM frequency of the patients must be ≤ 2 times a week when laxatives are not being taken;<br>(8) Patients must be capable of oral intake of drugs, food and beverages;<br>(9) Provision of written informed consent before participation.
Exclusion Criteria	(1) Patients diagnosed with clinically significant abnormal defecation due to structural abnormalities of the gastrointestinal tract and other tissues related to gastrointestinal tract (not including OIC): inflammatory bowel disease, irritable bowel syndrome, rectal prolapse, gastrointestinal obstruction, peritoneal metastasis, or peritoneal tumor at the time of enrollment;<br>(2) Patients with a history of gastrointestinal tract operation, abdominal operation, or abdominal adhesion within one month prior to screening; history of intestinal obstruction within three months prior to screening;<br>(3) Diagnosis of active diverticular disease; or severe hemorrhoid; or anal fissure; or artificial rectum or anus;<br>(4) Patients with an intraperitoneal catheter or a feeding tube;<br>(5) Diagnosis of pelvic disorder which are considered to have obvious effects on the intestinal transport of feces (such as uterine prolapse ≥degree 2, uterine fibroids [located in the posterior of the uterus with a diameter ≥ 5 cm] affecting bowel movement);<br>(6) Patients that are being treated with a new cancer chemotherapy, which had never been administered in the past, within 14 days of the screening or are scheduled to receive such therapy during the study;<br>(7) Patients that received radiotherapy within 28 days of the screening or are scheduled to receive such therapy during the study;<br>(8) Patients that underwent a surgery or intervention that is considered to have an obvious effect on the gastrointestinal functions within 28 days of the screening or

© 2023 Wang W et al. *JAMA Network Open.*

Continued

Exclusion Criteria	are scheduled to receive surgery or intervention which is considered to have obvious effects on the gastrointestinal functions during the study, or scheduled to receive surgery or intervention which will be anticipated to prevent the patients from completing the trial;<br>(9) Patients with uncontrolled hyperthyroidism, severe hypertension, heart disease, systematic infection or blood coagulation disorders (hypercoagulation status or hemorrhagic tendency) at the time of study inclusion;<br>(10) Patients that consumed >4 additional opioid doses per day, for breakthrough pain, for more than 3 days during the baseline period, or if their maintenance opioid dosing regimen was modified during this period;<br>(11) Patients with severe cancerous pain (e.g., typical average daily pain intensity rating of 7 to 10 on a numerical rating scales (NRS; 0 [no pain] to 10 [the worst pain possible]) after the use of routine dose and frequency of opioids) refractory to opioid therapy;<br>(12) Patients with a history of opioid discontinuation due to severe adverse events or patients that are expected to discontinue opioid use due to the potential risk of adverse events;<br>(13) Patients that received an opioid receptor antagonist within one month of the screening, or those who are scheduled to receive such therapy during the study;<br>(14) Patients with a history of nerve neurolysis;<br>(15) Patients with severe cognitive impairment, aphasia, or psychiatric disorders; abdominal aortic aneurysm; hepatomegaly(liver span > 14cm at the mid-clavicular line by ultrasound examination); or splenomegaly (spleen length [cranial to caudal] > 13cm by ultrasound examination);<br>(16) Patients that have received acupuncture within three months of the screening;<br>(17) Other patients who are considered ineligible for the study by the investigator on the basis of concomitant therapy and medical findings.

© 2023 Wang W et al. *JAMA Network Open.*

eFigure. Methods: Location of Acupoints for Both Study Arms

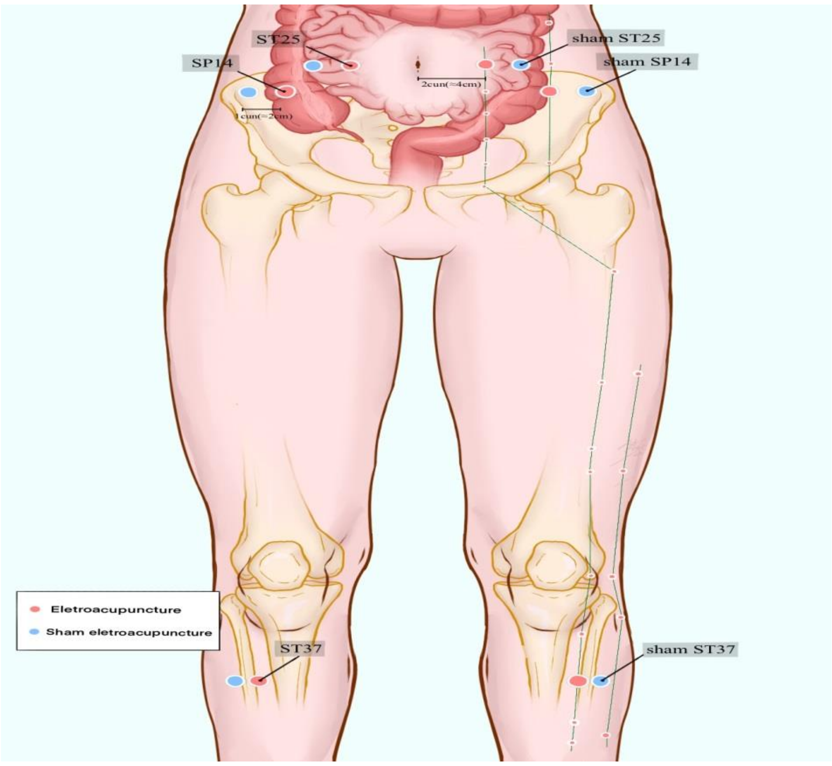

Bilateral acupoints of Tianshu (ST25), Fujie (SP14), and Shangjuxu (ST37) were used in the electroacupuncture group. Localization of these acupoints were based on the National Standard of the People's Republic of China (GB/T 12346–2006). [2]

Tianshu (ST25): on the upper abdomen, 2 B-cun lateral to the center of the umbilicus.

Fujie (SP14): on the lower abdomen, 1.3 B-cun inferior to the center of the umbilicus, 4 B-cun lateral to the anterior median line.

Shangjuxu (ST37): on the anterior aspect of the leg, on the line connecting Dubi (ST35) with Jiexi(ST41), 6 B-cun inferior to ST35.

Bilateral sham acupoints of Tianshu (ST25), Fujie (SP14), and Shangjuxu (ST37) were used in the sham electroacupuncture group. The sham ST25 and SP14 are situated 2 cm horizontally outward from ST25 and SP14 respectively. Sham ST37 are located outward from ST37 in the middle of stomach and gallbladder channel.

© 2023 Wang W et al. *JAMA Network Open.*

eAppendix. Methods: Multiple Imputation and Sensitivity Analyses

Multiple imputation methods for the primary outcome.

We had 8 cases with missing data. Multiple imputation was used to impute missing values under the missing-at random assumption (MAR). Specifically, 100 imputed data sets were generated using the fully conditional specification method with the number of iterations set to 10 for the following variables: group (electroacupuncture and sham electroacupuncture), and response variable (responders: yes, no). After multiple imputation, each of the hundred multiple imputation datasets was analyzed by generalized linear model. The overall estimates were calculated using Rubin's rules. The multiple imputation procedure (PROC MI) in SAS, version 9.4 was used. (Detailed in Table 2)

The analysis of the primary outcome was repeated using 4 analytical approaches.

First, a control-based pattern imputation model[3] was assessed whether the primary outcome was robust to departure from MAR. More specifically, an imputation model for the missing observations in the acupuncture group was constructed from the observed data in the sham electroacupuncture group rather than the electroacupuncture group. Parallel to the primary analysis based on MAR, we used a similar method with such an imputed data set to show the robustness of the final results. SAS PROC MI with the MNAR statement was used. The results were robust to departure from MAR. (Detailed in model 1 of eTable 4)

The second approach was conducted among patients who provided complete data (47 in EA, 45 in SA).

The third approach was conducted by adding the acupuncturist variable as a fixed effect to account for clustering by acupuncturists. [4]

The fourth approach was performed after deleting study subjects who responded that they were allocated to the sham arm.

© 2023 Wang W et al. *JAMA Network Open.*

eTable 2. Results: Patients' Expectations About the Effectiveness of Acupuncture at Baseline

	**Electroacupuncture (N=50)**	**Sham electroacupuncture (N=50)**
Do you think acupuncture will be effective in treating diseases in general? n (%)		
Yes	38 (76.0)	35 (70.0)
No	0	0
Unclear	12 (24.0)	15 (30.0)
Do you think acupuncture will be effective for opioid-induced constipation? n (%)		
Yes	31 (62.0)	29 (58.0)
No	0	0
Unclear	19 (38.0)	21 (42.0)

© 2023 Wang W et al. *JAMA Network Open.*

eTable 3. Results: Compliance Data at Week 8[a]

	**Electroacupuncture**	**Sham electroacupuncture**	**Total**
Number of treatment sessions received, mean (SD)	21.7 (4.1)	21.0 (6.2)	21.4 (5.3)
Patients (percentage) received at least 20 sessions of treatment (compliance rates ≥ 80%), n (%)	44 (88.0)	42 (84.0)	82 (82.0)

[a] Eight of 100 participants (3 in the electroacupuncture group and 5 in the sham electroacupuncture group) did not complete the 8-week treatment due to various reasons.

© 2023 Wang W et al. *JAMA Network Open.*

eTable 4. Sensitivity Analyses for the Primary Outcome

Approaches	Electroacupuncture	Sham electroacupuncture	Difference (95%CI)	*P* Value
Method 1	38.4 (24.8 to 52.0)	9.7 (0.8 to 18.6)	28.7 (12.5 to 44.8)	<0.001
Method 2	19/47 (40.4)	4/45 (8.9)	31.5 (15.2 to 47.9)	<0.001
Method 3	19/47 (40.4)	4/45 (8.9)	32.9 (13.9 to 51.8)	<0.001
Method 4	18/46 (39.1)	2/22 (9.1)	30.0 (11.5 to 48.6)	0.002

© 2023 Wang W et al. *JAMA Network Open.*

eTable 5. Results: Subgroup Analyses[a]

	**Electroacupuncture**	**Sham electroacupuncture**	**Difference (95%CI)**	***P* value**
**Number of patients stratified by opioid dose[b]**				
30-100 mg group	12/34 (35.3)	3/35 (8.6)	26.7 (8.2 to 45.3)	0.005
>100 mg group	7/13 (53.9)	1/10 (10.0)	43.9 (11.0 to 76.7)	0.009
**Number of patients stratified by the type of primary cancer[c]**				
Lung cancer	7/16 (43.8)	2/20 (10.0)	33.8 (6.1 to 61.4)	0.02
Non-lung cancer	12/31 (38.7)	2/25 (8.0)	30.7 (10.5 to 50.9)	0.003

[a] Values are reported as no./total no. (%) unless otherwise indicated.

[b] A pre-specified subgroup analysis based on the baseline opioid dose strata was conducted for the primary outcome.

[c] A post hoc subgroup analysis based on the baseline primary cancer type was conducted for the primary outcome.

© 2023 Wang W et al. *JAMA Network Open.*

eTable 6. Results: Weekly SBMs

Week	Electroacupuncture			Sham electroacupuncture	
	n	Weekly SBMs, mean (95%CI)		n	Weekly SBMs, mean (95%CI)
-1	50	1.6 (1.5 to 1.8)		50	1.5 (1.4 to 1.7)
1	50	2.2 (1.9 to 2.5)		48	1.9 (1.6 to 2.2)
2	50	2.45 (2.2 to 2.8)		48	2.0 (1.7 to 2.3)
3	49	2.6 (2.3 to 2.8)		45	2.2 (1.9 to 2.5)
4	48	2.8 (2.6 to 3.1)		45	2.0 (1.8 to 2.3)
5	47	3.0 (2.7 to 3.3)		45	2.2 (1.9 to 2.5)
6	47	2.9 (2.7 to 3.2)		45	2.3 (2.0 to 2.6)
7	47	3.1 (2.8 to 3.4)		45	2.2 (1.9 to 2.6)
8	47	3.2 (3.0 to 3.5)		45	2.3 (2.0 to 2.7)
13	46	2.4 (2.1 to 2.6)		41	1.7 (1.5 to 1.9)
14	46	2.1 (1.9 to 2.4)		41	1.9 (1.7 to 2.1)
15	46	2.4 (2.0 to 2.6)		41	1.8 (1.6 to 2.1)
16	46	2.3 (2.0 to 2.6)		41	1.8 (1.5 to 2.0)

SBM = spontaneous bowel movement.

© 2023 Wang W et al. *JAMA Network Open.*

eTable 7. Results: PAC-QOL Total and Subscale Scores[a]

Variable	Electroacupuncture (n=50)	Sham electroacupuncture (n=50)	Difference (95% CI)	*P* Value
PAC-QOL total score at baseline, mean (SD) [b]	2.2 (0.8)	2.3 (0.8)	—	—
Physical discomfort subscale score	2.1 (0.9)	2.2 (0.9)	—	—
Psychosocial discomfort subscale score	1.5 (0.8)	1.6 (0.8)	—	—
Worries/concerns subscale score	2.3 (1.0)	2.3 (0.9)	—	—
Satisfaction subscale score	3.0 (0.9)	3.2 (0.8)	—	—
Change in PAC-QOL total score, mean (95% CI)				
Week 8	-0.6 (-0.8 to -0.5)	-0.4 (-0.5 to -0.2)	-0.3 (-0.5 to -0.1)	0.01
Week 16	-0.4 (-0.5 to -0.2)	-0.1 (-0.3 to 0.1)	-0.3 (-0.5 to -0.03)	0.03
Change in Physical discomfort subscale score, mean (95% CI)				
Week 8	-0.7 (-0.8 to -0.5)	-0.5 (-0.7 to -0.3)	-0.2 (-0.5 to 0.1)	0.13
Week 16	-0.4 (-0.6 to -0.2)	-0.1 (-0.3 to 0.1)	-0.3 (-0.6 to 0.02)	0.07
Change in Psychosocial discomfort subscale score, mean (95% CI)				
Week 8	-0.4 (-0.6 to -0.3)	-0.2 (-0.3 to -0.1)	-0.2 (-0.4 to -0.01)	0.04
Week 16	-0.3 (-0.4 to -0.1)	-0.03 (-0.2 to 0.1)	-0.3 (-0.5 to -0.04)	0.02
Change in Worries/concerns subscale score, mean (95% CI)				
Week 8	-0.6 (-0.8 to -0.5)	-0.3 (-0.5 to -0.1)	-0.3 (-0.5 to -0.1)	0.01
Week 16	-0.4 (-0.5 to -0.2)	-0.1 (-0.2 to 0.1)	-0.3 (-0.6 to -0.03)	0.03

© 2023 Wang W et al. *JAMA Network Open.*

Continued

**Variable**	**Electroacupuncture (n=50)**	**Sham electroacupuncture (n=50)**	**Difference (95% CI)**	***P* Value**
Change in Satisfaction subscale score, mean (95% CI)				
Week 8	-1.0 (-1.2 to -0.7)	-0.6 (-0.9 to -0.4)	-0.3 (-0.6 to 0.01)	0.05
Week 16	-0.5 (-0.8 to -0.3)	-0.3 (-0.6 to -0.1)	-0.2 (-0.5 to 0.2)	0.32

[a] Three patients in the electroacupuncture group and 5 patients in the sham electroacupuncture group did not complete 8-week treatment over weeks 1-8. One patient in the electroacupuncture group and 4 participants in sham electroacupuncture group dropped out over weeks 9-16.

[b] PAC-QOL scores are based on a 5-point Likert scale from 0 to 4. Minimally clinically important difference: 1.[5] A lower score indicates better quality of life.

© 2023 Wang W et al. *JAMA Network Open.*

eTable 8. Results: Patients' Global Improvement Assessment, n (%)

	Week 8			Week 16	
	**Eectroacupuncture (N= 50)**	**Sham electroacupuncture (N= 50)**		**Eectroacupuncture (N= 50)**	**Sham electroacupuncture (N= 50)**
Markedly improved	13 (27.7)	3 (6.7)		8 (17.4)	0
Moderately improved	21 (44.7)	4 (8.9)		6 (13.0)	1 (2.4)
Slightly improved	10 (21.3)	30 (66.7)		13 (28.3)	10 (24.4)
No change	2 (4.3)	8 (17.8)		3 (6.5)	19 (46.3)
Slightly worse	0	0		6 (13.0)	1 (2.4)
Moderately worse	1 (2.1)	0		6 (13.0)	5 (12.2)
Markedly worse	0	0		4 (8.7)	5 (12.2)
Missing	3	5		4	9

© 2023 Wang W et al. *JAMA Network Open.*

eTable 9. Results: Blinding Data and Their Association With the Primary Outcome by the Respective Group[a]

Assignment	Guess	
	**Conventional electroacupuncture**	**Sham electroacupuncture**
Electroacupuncture, n (%)	n=46	n=1
Overall responder	18 (39.1)	1 (100)
Non-responder	28 (60.9)	0
Sham electroacupuncture, n (%)	n=22	n=23
Overall responder	2 (9.1)	2 (8.7)
Non-responder	20 (90.9)	21 (91.3)

[a] Values were reported as no./total no. (%) unless otherwise indicated. Answers from 3 patients in the electroacupuncture group and 5 patients in the sham electroacupuncture group were not recorded due to staff negligence.

Before treatment, we told patients that they had a 50% chance of receiving conventional electroacupuncture with a deeper needle insertion or minimal electroacupuncture with a superficial needle penetration. Both conventional electroacupuncture and minimal electroacupuncture may all have some effects on opioid induced constipation. Both treatments used a relatively small electric intensity, and patients may or may not feel the stimulation during treatment because of the relatively weak electrical stimulation and the tolerance of the human body. To assess the success of blinding, within 5 minutes after any treatment session at week 8, patients were asked to guess whether they had received conventional electroacupuncture in the previous weeks (yes or no).

© 2023 Wang W et al. *JAMA Network Open.*

eTable 10. Results: Change From Baseline in NRS Score for Mean Cancer Pain Intensity and Worst Pain Intensity Within the Preceding Week

Variable	Electroacupuncture	Sham electroacupuncture	Difference (95%CI)	*P* Value
Change in NRS score for mean cancer pain intensity within the preceding week from baseline, mean (95%CI)				
Week 8[a]	-0.2 (-0.4 to 0.01)	-0.03 (-0.2 to 0.2)	-0.2 (-0.4 to 0.1)	0.20
Week 16[b]	-0.1 (-0.4 to 0.2)	0.1 (-0.2 to 0.4)	-0.2 (-0.6 to 0.2)	0.26
Change in NRS score for worst cancer pain intensity within the preceding week from baseline, mean (95%CI)				
Week 8[a]	-0.2 (-0.4 to 0.1)	-0.1 (-0.3 to 0.2)	-0.1 (-0.5 to 0.2)	0.50
Week 16[b]	0.03 (-0.4 to 0.4)	0.2 (-0.2 to 0.6)	-0.2 (-0.8 to 0.4)	0.49

NRS = numerical rating scale (0 [no pain] to 10 [the worst pain possible]).

[a] Three participants in the electroacupuncture group and 5 participants in the sham electroacupuncture group did not complete 8-week treatment over weeks 1-8. To obtain the value at week 8, the values at weeks 2, 4, 6, and 8 are summed and divided by 4.

[b] One participant in the electroacupuncture group and 4 participants in sham electroacupuncture group dropped out over weeks 9-16. Value at week 16 is a single value.

© 2023 Wang W et al. *JAMA Network Open.*

eTable 11. Results: Proportion of Patients With Change From Baseline in the Dose of Opioid Consumption, n (%)

	**Weeks 1-8**			**Weeks 9-16**	
	**Electroacupuncture**	**Sham electroacupuncture**		**Electroacupuncture**	**Sham electroacupuncture**
Remained unchanged	40 (85.1)	34 (75.6)		33 (71.7)	29 (70.7)
Participants with the opioid consumption increase ≥30%	6 (12.8)	7 (15.6)		10 (21.7)	9 (22.0)
Participants with the opioid consumption increase <30%	1 (2.1)	4 (8.9)		3 (6.5)	3 (7.3)
Participants with the opioid consumption decrease ≥30%	0	0		0	0
Participants with the opioid consumption decrease <30%	0	0		0	0
Missing	3	5		4	9

© 2023 Wang W et al. *JAMA Network Open.*

eReferences.

1. Lacy BE, Mearin F, Chang L, et al. Bowel disorders. *Gastroenterology*. 2016; 50(6): 1393-1407.

2. Standardization Administration of the People's Republic of China. GB/ T12346-2006, Nomenclature and Location of Acupuncture Points [S].2006.

3. Bohdana Ratitch Q, Montreal Q, Canada Michael O'Kelly Q, Dublin I. Implementation of pattern-mixture models using standard SAS/STAT procedures. 2011. http://pharmasug.org/proceedings/2011/SP/PharmaSUG-2011-SP04.pdf. Accessed April 21,2012.

4. Lee, K. J. Clustering by health professional in individually randomised trials[J]. *BMJ*. 2005, 330(7483):142-144.

5. Tack J, Camilleri M, Hale M, et al. Establishing Minimal Clinically Important Differences in Quality of Life Measures in Opioid-Induced Constipation. Clin Gastroenterol Hepatol. 2022; 20(4):855-863.

© 2023 Wang W et al. *JAMA Network Open.*
