# Supplementary material for: Effects of Electroacupuncture for Opioid-Induced Constipation in Patients With Cancer in China: A Randomized Clinical Trial
Source: JAMA Netw Open. 2023 Feb 22;6(2):e230310. doi: 10.1001/jamanetworkopen.2023.0310 (PMC9947731; doi:10.1001/jamanetworkopen.2023.0310)
Supplement: Supplement 3. — Data Sharing Statement [file jamanetwopen-e230310-s003.pdf]

Data Sharing Statement

Wang. Effects of Electroacupuncture for Opioid-Induced Constipation in Patients With Cancer in China. *JAMA Netw Open*. Published February 22, 2023.
doi:10.1001/jamanetworkopen.2023.0310

Data
Data available: Yes
Data types: Participant data with identifiers
How to access data: A formal request with a methodologically sound proposal should be sent to Dr. Zhishun Liu (e-mail, zhishunjournal@163.com).
When available: beginning date: 12-31-2023

Supporting Documents
Document types: Statistical/analytic code
How to access documents: A formal request with a methodologically sound proposal should be sent to Dr. Zhishun Liu (e-mail, zhishunjournal@163.com).
When available: beginning date: 12-31-2023

Additional Information
Who can access the data: The data and documents will be available to researchers whose proposal has been approved for a specified purpose.
Types of analyses: A formal request with a methodologically sound proposal.
Mechanisms of data availability: Researchers whose proposal has been approved will be required to sign a data access agreement
Any additional restrictions: none
